# Nuclear Translocation of S100A9 Triggers Senescence of Human Amnion Fibroblasts by De‐Repressing LINE1 Via Heterochromatin Erosion at Parturition

**DOI:** 10.1002/advs.202414682

**Published:** 2025-04-02

**Authors:** Fan Zhang, Meng‐Die Li, Fan Pan, Wen‐Jia Lei, Yang Xi, Li‐Jun Ling, Leslie Myatt, Kang Sun, Wang‐Sheng Wang

**Affiliations:** ^1^ Center for Reproductive Medicine Ren Ji Hospital Shanghai Jiao Tong University School of Medicine Shanghai 200135 P.R. China; ^2^ Shanghai Key Laboratory for Assisted Reproduction and Reproductive Genetics Shanghai 200135 P. R. China; ^3^ Department of Obstetrics Shanghai First Maternity and Infant Hospital Tongji University School of Medicine Shanghai 200040 P. R. China; ^4^ Central Laboratory Shanghai Xuhui Central Hospital Zhongshan‐Xuhui Hospital Fudan University Shanghai 200031 P. R. China; ^5^ Department of Obstetrics and Gynecology Oregon Health & Science University Portland OR 97239 USA

**Keywords:** fetal membranes, heterochromatin, LINE1, parturition, S100A9, senescence

## Abstract

Aging of the fetal membranes participates in labor onset. However, the underlying mechanism is poorly understood. Here, we identify that the classical secretory protein S100 calcium‐binding protein A9 (S100A9), upon de‐phosphorylation at Thr 113, translocates to the nuclei of amnion fibroblasts of the human fetal membranes, where S100A9 causes heterochromatin erosion via segregation of heterochromatin maintenance proteins, resulting in Long Interspersed Nuclear Element‐1 (LINE1) de‐repression at parturition. Increased LINE1 retrotransposition further activates the type I interferon response via the cGAS‐STING pathway, thereby leading to amnion fibroblast senescence with consequent increased secretion of components associated with senescence‐associated secretory phenotype. Mouse studies show that intra‐amniotic injection of vector specifically expressing S100A9 in the nucleus induces preterm birth along with LINE1 de‐repression and increased cellular senescence in the fetal membranes, which is blocked by inhibition of LINE1 reverse‐transcription. Together, these findings highlight that nuclear‐translocated S100A9 acts as a heterochromatin disruptor to de‐repress LINE1 which subsequently triggers amnion fibroblast senescence at parturition.

## Graphical abstract

This study shows that the classical secretory protein S100 calcium‐binding protein A9 (S100A9) can translocate to the nucleus upon de‐phosphorylation at Thr 113 in human amnion fibroblasts at parturition, where S100A9 induces heterochromatin erosion through segregation of the heterochromatin maintenance protein, resulting inLong Interspersed Nuclear Element‐1 (LINE1) de‐repression. Increased LINE1 retrotransposition causes cellular senescence via activation of the cGAS‐STING‐type I interferon response pathway in human amnion fibroblasts.

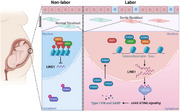

## 1 Introduction

Cellular senescence is an irreversible biological process that progresses with age, heralding the end of lifespan in almost all multicellular species.^[^ ^1^ ^]^ The placenta and fetal membranes are unique temporary organs in pregnancy. They fulfill their mission once pregnancy terminates. Accumulating evidence indicates that toward the end of gestation cells in the placenta and fetal membranes undergo senescence, a process which is believed to be an integral event of labor onset.^[^ ^2^ ^]^ Cellular senescence and senescence‐associated secretory phenotype (SASP) provoke inflammatory reactions in these tissues leading to overwhelming production of pro‐labor factors.^[^ ^3^ ^]^ In the fetal membranes, cellular senescence may also weaken the tensile strength of the membranes thus enabling membrane rupture which further enhances the production of pro‐labor factors.^[^ ^3e^ ^]^ Therefore, understanding the cause of cellular senescence in the fetal membranes is of utmost importance for elucidation of the mechanism whereby the fetal membranes initiate parturition in humans, which may help develop effective strategies for the prevention of preterm birth, a major cause of perinatal mortality and morbidity.^[^ ^4^ ^]^

Among the identified causes of cellular senescence, epigenomic instability, especially heterochromatin disorganization, is emerging as a prominent trigger.^[^ ^5^ ^]^ Heterochromatin is defined as a densely packed chromatin, rendering access of DNA and RNA polymerases to the associated gene difficult, thereby preventing DNA replication and gene transcription.^[^ ^6^ ^]^ Maintenance of heterochromatin is normally achieved through enrichment of trimethylated histone H3 at lysine 9 (H3K9me3) as well as heterochromatin maintenance proteins, such as CBX5 (Chromobox protein homolog 5, also known as heterochromatin protein 1α, HP1α), KRAB‐associated protein 1 (KAP1) and the nucleosome remodeling and deacetylase (NuRD) complex.^[^ ^6^ ^]^ Heterochromatin is crucial for silencing transposable elements (TEs), especially Long Interspersed Nuclear Element‐1 (LINE‐1), which are the most abundant and only active autonomous retrotransposon in humans, accounting for nearly 17% of the human genome.^[^ ^7^ ^]^ Tight repression of LINE‐1 expression is essential for the prevention of DNA damage, which is normally required to maintain genomic integrity.^[^ ^7e^ ^]^ Emerging evidence indicates that increased LINE1 transcription and retrotransposition as a result of heterochromatin erosion are associated with cellular senescence in a variety of non‐gestational tissues.^[^ ^8^ ^]^ It is recognized that increased LINE1 transcription and subsequent retrotransposition trigger cellular senescence through activation of the innate immune response such as the type I interferon (IFN) response.^[^ ^8^ ^]^ However, whether the de‐repression of LINE1 as a result of heterochromatin disorganization participates in cellular senescence of the fetal membranes remains unknown. If so, it is crucial to identify the inducer of heterochromatin erosion in the fetal membranes so that novel therapeutic targets can be developed for the treatment of preterm premature rupture of membranes and preterm birth.

S100 calcium‐binding protein A9 (S100A9, also known as MRP14) is a member of the S100 family of calcium‐binding proteins, which is normally secreted to the extracellular space as a cytokine or as a protein of damage‐associated molecular patterns in response to inflammatory stimuli, tissue damage, and stress, etc.^[^ ^9^ ^]^ By re‐examination of our previously published transcriptomic data,^[^ ^10^ ^]^ we found that the abundance of S100A9 was significantly increased in the amnion layer of the human fetal membranes at parturition. Serendipitously, we found that S100A9 was localized in the nuclei of mesenchymal fibroblasts of the human amnion (human amnion fibroblasts, hAFs), a distinct cell type of the amnion layer that not only provides pro‐labor factors but also extracellular matrix (ECM) components.^[^ ^11^ ^]^ More intriguingly, we found that S100A9 could translocate to the nucleus upon de‐phosphorylation at Thr 113 in hAFs, where S100A9 caused heterochromatin disruption through segregation of heterochromatin maintenance proteins such as CBX5, thus leading to LINE1 de‐repression. LINE1 retrotransposition, in turn, stimulated the type I IFN response through activation of the cGAS (cyclic GMP‐AMP synthase)‐STING (stimulator of interferon genes) signaling pathway, resulting in cellular senescence in hAFs. Mouse studies showed that intra‐amniotic administration of vector specifically expressing S100A9 in the nucleus induced preterm birth along with increased LINE1 activation and cellular senescence in the fetal membranes, which lent further support for the crucial role of nuclear S100A9 in the induction of cellular senescence in the fetal membranes at parturition. Here, we described the identification of this novel crucial role of nuclear‐translocated S100A9 in the induction of cellular senescence in the fetal membranes at parturition.

## 2 Results

### 2.1 Increased S100A9 in the Nuclei of hAFs at Term and Preterm Labor

**Figure 1:**
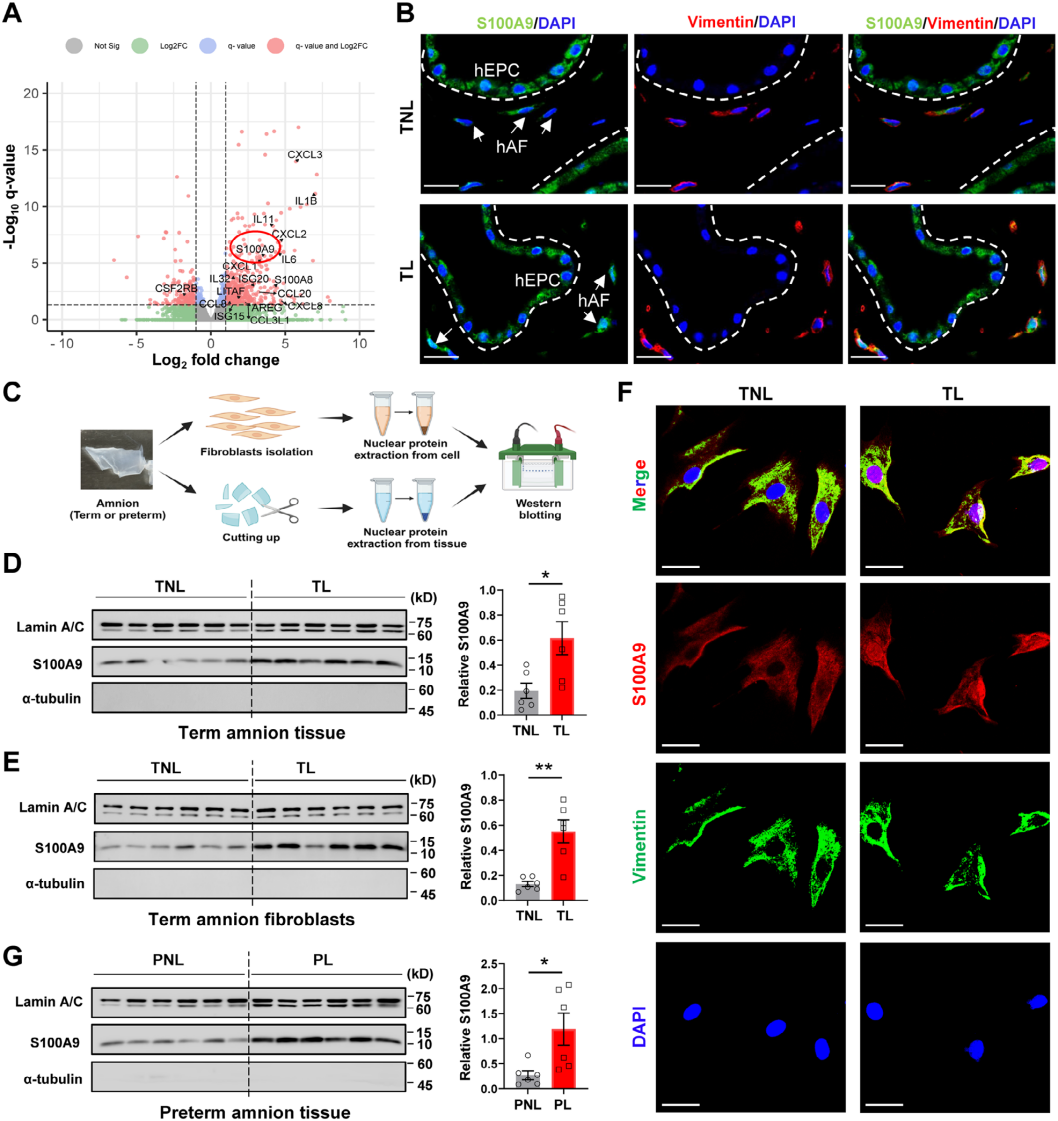
Increased nuclear S100A9 in hAFs at parturition. A) Volcano plot of transcriptomic sequencing data displaying the differentially expressed genes of the human amnion in the TL group (n = 3) as compared to the TNL group (n = 3). TL, term labor; TNL, term no labor. B) Representative images of immunofluorescence staining showing intense S100A9 staining (green color) in the nuclei of hAFs from the TL amnion. n = 3. White arrows indicate hAFs, which are positive for vimentin staining (red color), a classical mesenchymal fibroblast marker. Nuclei were counterstained with DAPI (blue color). Scale bars, 25 µm. C) Diagram illustrating the procedure of nuclear protein extraction from human amnion tissue and isolated hAFs. D) Western blot analysis showing the difference of nuclear S100A9 protein abundance in the human amnion between TL (n = 6) and TNL (n = 6) groups. The left panel is the Western blot and the right panel is the average data. α‐tubulin and Lamin A/C were used as internal controls for cytoplasmic and nuclear protein, respectively. E) Western blotting analysis showing the difference of S100A9 protein abundance in isolated hAFs between TL (n = 6) and TNL (n = 6) groups. The left panel is the Western blot and the right panel is the average data. α‐tubulin and Lamin A/C were used as internal controls for cytoplasmic and nuclear protein, respectively. F) Representative images of immunofluorescence staining showing intense S100A9 staining (red color) in the nuclei of hAFs isolated from the TL amnion. n = 3. hAFs were positive for vimentin staining (green color). Nuclei were counterstained with DAPI (blue color). Scar bar, 25 µm. G) Western blot analysis showing the difference of nuclear S100A9 protein abundance in the human amnion between PL (n = 6) and PNL (n = 6) groups. The left panel is the Western blot and the right panel is the average data. α‐tubulin and Lamin A/C were used as internal controls for cytoplasmic and nuclear protein, respectively. PL, preterm labor; PNL, preterm no labor. Data are mean ± SEM. Two‐tailed unpaired Student's t‐test (D, E, G). *p < 0.05, **p < 0.01.

Reanalysis of our previously published transcriptomic sequencing data (NCBI GEO accession number GSE166453)^[^ ^10^ ^]^ revealed a significant increase in *S100A9* mRNA in the amnion layer of the fetal membranes obtained from pregnant women undergoing spontaneous labor at term (designated as term labor, TL) when compared with that obtained from pregnant women undergoing elective cesarean section without labor at term (designated as term no labor, TNL) (**Figure** **1**A). The increase in S100A9 in the TL amnion was subsequently confirmed at mRNA and protein levels with quantitative real‐time polymerase chain reaction (qRT‐PCR), Western blotting, and enzyme immunoassay (ELISA) respectively (Figure S1A–C, Supporting Information). Intriguingly, despite S100A9 being a classical secretory protein,^[^ ^9b^ ^]^ immunofluorescent and immunohistochemical staining of the human amnion showed an intense distribution of S100A9 in the nuclei of hAFs in the TL group (Figure 1B; Figure S1D, Supporting Information). However, this phenomenon was not observed in epithelial cells of the human amnion (hEPCs). We further compared the abundance of nuclear S100A9 between the TL and TNL groups in the nuclear protein extracted from the TL and TNL amnion as well as from hAFs isolated from the TL and TNL amnion with Western blotting, which revealed an increased abundance of nuclear S100A9 in both the amnion tissue and hAFs at parturition (Figure 1C–E). Immunofluorescent staining also showed an intense staining of S100A9 in the nuclei of hAFs isolated from the TL amnion (Figure 1F). The identity of hAFs in the amnion and isolated hAFs was confirmed with immunofluorescent staining for vimentin, a mesenchymal cell marker (Figure 1B,F; Figure S1E, Supporting Information). Further, a significant increase in nuclear S100A9 abundance was also observed in the amnion obtained from spontaneous preterm labor with no indication of infection (designated as preterm labor, PL) as compared to preterm elective cesarean section without labor (designated as preterm no labor, PNL) (Figure 1G). These data indicate that S100A9 nuclear translocation in hAFs may play a role in both term and preterm labor.

### 2.2 Correlation of Cellular Senescence with Nuclear S100A9 in hAFs at Parturition

**Figure 2:**
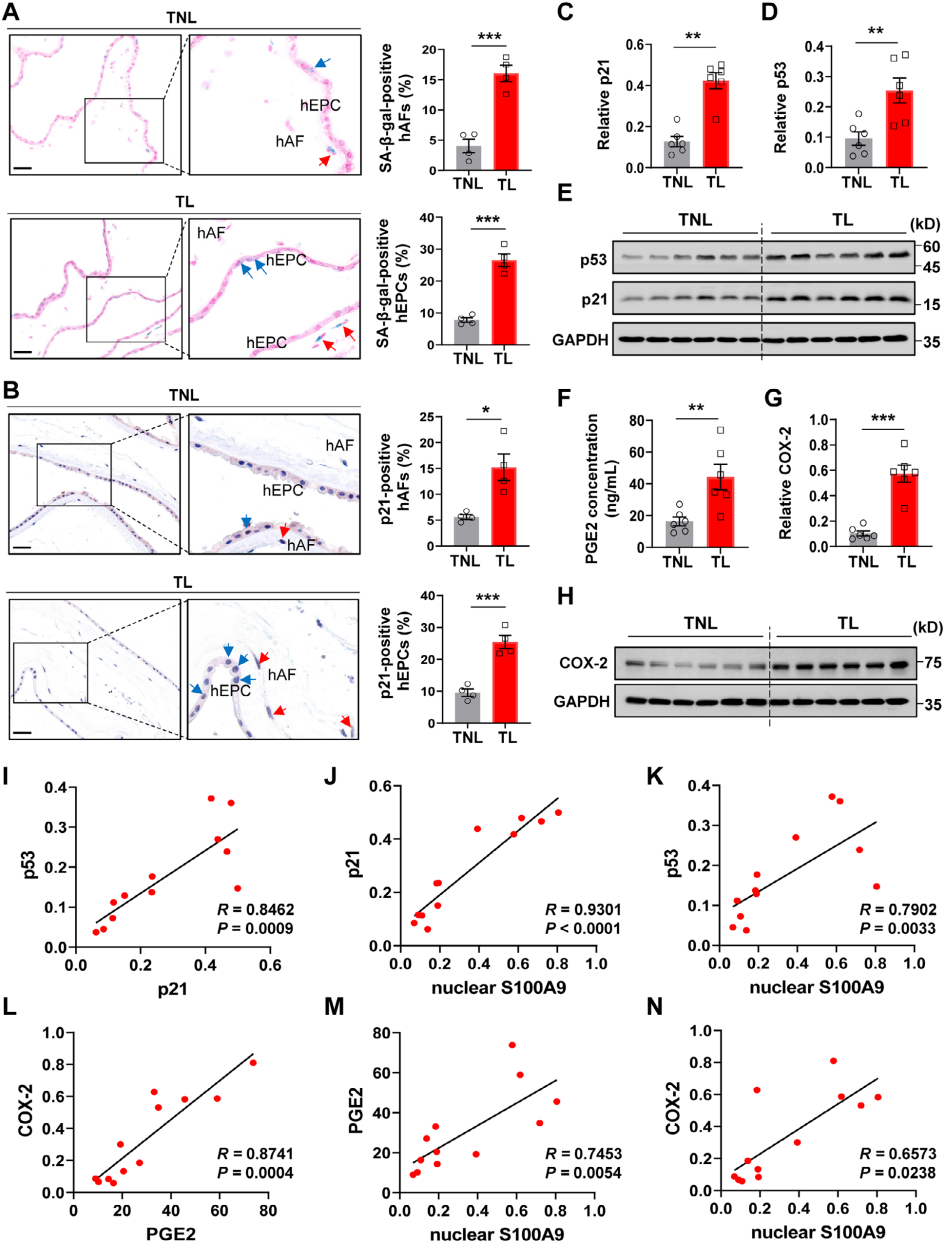
Increased cellular senescence and PGE2 production in hAFs at parturition. A) SA‐β‐gal staining showing an increased percentage of cells positive for SA‐β‐gal staining (blue color) in the human amnion collected from TL (n = 4) compared to TNL (n = 4). Nuclei were counterstained with nuclear fast red (red color). Red and blue arrows indicate SA‐β‐gal‐positive hAFs and hEPCs respectively. The left panel is the representative image and the right panel is the average data. Scale bars, 50 µm. B) Immunohistochemical staining of p21 showing an increased percentage of cells positive for p21 (red color) in the human amnion collected from TL (n = 4) compared to TNL (n = 4). The tissue section was counterstained with hematoxylin (blue color). Red and blue arrows indicate p21‐positive hAFs and hEPCs respectively. Left panel is the representative image and the right panel is the average data. Scale bars, 50 µm. C–E) Western blot analysis showing increased p21 and p53 protein abundance in hAFs isolated from TL (n = 6) compared to TNL (n = 6). (E) is the Western blot and (C‐D) is the average data. F) ELISA showing increased PGE2 concentration in the conditioned culture medium of hAFs isolated from TL (n = 6) compared to that from TNL (n = 6). G–H) Western blot analysis showing increased COX‐2 protein abundance in hAFs isolated from TL (n = 6) compared to that from TNL (n = 6). (H) is the Western blot and (G) is the average data. I) Spearman analysis showing a positive correlation between p21 and p53 in isolated hAFs. n = 12. J,K) Spearman analysis showing a positive correlation between nuclear‐localized S100A9 and p21 (J) or p53 (K) in isolated hAFs. n = 12. L) Spearman analysis showing a positive correlation between COX‐2 and PGE2 in isolated hAFs. n = 12. M,N) Spearman analysis showing a positive correlation between nuclear‐localized S100A9 and PGE2 (M) or COX‐2 (N) in isolated hAFs. n = 12. Data are mean ± SEM. Two‐tailed unpaired Student's t‐test (A, B, D, F, G), or Mann–Whitney U test (C). *p < 0.05, **p < 0.01, ***p < 0.001.

Previous studies have shown that cellular senescence of the amnion at parturition is largely an epithelial phenomenon.^[^ ^2b–d^ ^]^ However, it is not known whether hAFs also undergo cellular senescence at parturition. By staining the amnion with senescence‐associated β‐galactosidase (SA‐β‐gal) and p21, a classical marker of cellular senescence,^[^ ^1^, ^12^ ^]^ we observed that the proportion of SA‐β‐gal‐ and p21‐positive cells were significantly higher in both hEPCs and hAFs of the TL amnion versus TNL amnion (**Figure** **2**A,B). Cellular senescence in hAFs was further confirmed with Western blotting, which showed significantly increased p53 and p21 protein abundance in hAFs isolated from the TL amnion versus TNL amnion (Figure 2C–E). Additionally, ELISA assay showed that the level of prostaglandin E2 (PGE2), a well‐recognized pro‐labor eicosanoid that is crucial for cervical ripening and myometrial contraction in parturition,^[^ ^3a^ ^]^ was significantly higher in hAFs isolated from the TL amnion than that from the TNL amnion (Figure 2F). Correspondingly, the inducible cyclooxygenase‐2 (COX‐2, encoded by *PTGS2*), a rate‐limiting enzyme in prostaglandin synthesis, was also significantly increased in hAFs isolated from the TL amnion as compared to that from the TNL amnion (Figure 2G,H). Moreover, correlation analysis showed that the abundance of COX‐2, PGE2, p21, p53, an upstream stimulator of p21, and nuclear S100A9 correlated positively with each other in hAFs (Figure 2I‐N; Figure S1F–I, Supporting Information). These data suggest that hAFs, in addition to hEPCs, also undergo cellular senescence at parturition. Moreover, cellular senescence and increased PGE2 production in hAFs may be associated with nucleus‐translocated S100A9.

### 2.3 Induction of Cellular Senescence by Nuclear S100A9 in hAFs

**Figure 3:**
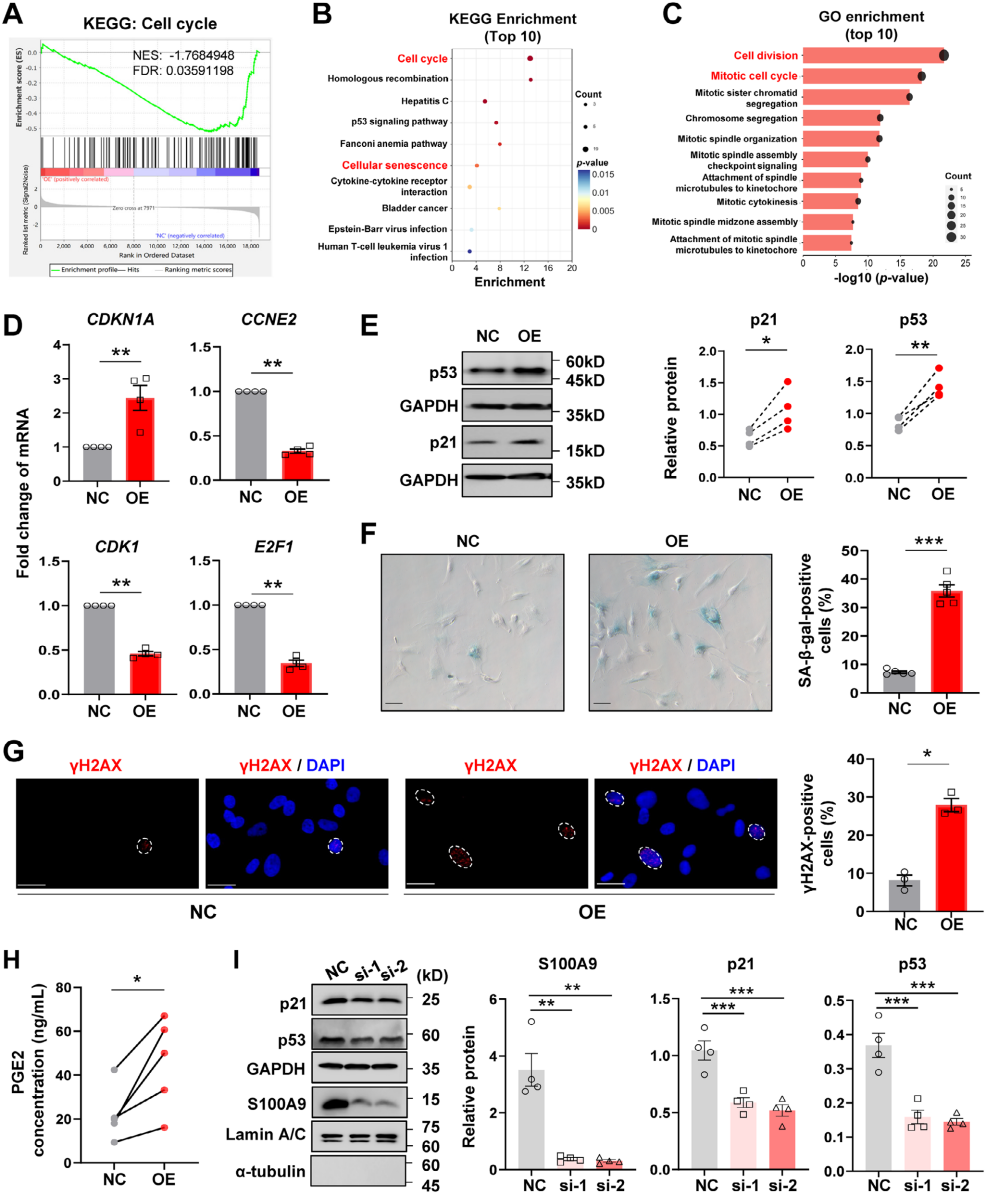
Induction of cellular senescence by nuclear S100A9 in hAFs. A) Gene Set Enrichment Analysis (GSEA) in Kyoto encyclopedia of genes and genomes (KEGG) enrichment showed that the cell cycle pathway was downregulated in hAFs by overexpression of S100A9 in the nuclei (n = 3 per group). NES, normalized enrichment scores; FDR, false discovery rate; NC, control with transfection of empty vector; OE, overexpression of S100A9 in the nuclei. B,C) KEGG (B) and Gene ontology (GO) biological process (C) analyses of the transcriptomic sequencing data obtained from hAFs with nuclear S100A9 overexpression (n = 3 per group) showing the pathways that the differentially expressed genes were enriched in. D) qRT‐PCR analysis showing the difference of *CDKN1A, CCNE2, CDK1*, and *E2F1* mRNA abundance between hAFs with and without nuclear S100A9 overexpression. n = 4. E) Western blot analysis showing the difference of p21 and p53 protein abundance between hAFs with and without nuclear S100A9 overexpression. n = 4. The left panel is the representative blot and the right panel is the average data. F) SA‐β‐gal staining showing the percentage of SA‐β‐gal positive cells (blue color) in cultured hAFs with and without nuclear S100A9 overexpression. The left panel is the representative image and the right panel is the average data. Scale bars, 25 µm. n = 5. G) Immunofluorescence staining of γH2AX, a sensitive marker of DNA damage response, showing the percentage of γH2AX‐positive cells (red color) in hAFs with and without nuclear S100A9 overexpression. Nuclei were counterstained with DAPI (blue color). Dashed lines indicate the nuclear boundaries of the γH2AX‐positive (> 2 red dots) cells. The left panel is the representative image and the right panel is the average data. Scale bars, 25 µm. n = 3. H) The difference of PGE2 concentrations between the conditioned culture medium obtained from hAFs with and without nuclear S100A9 overexpression as measured with ELISA. n = 5. I) Western blot analysis showing the protein abundance of p21 and p53 in hAFs with or without siRNA‐mediated knockdown of *S100A9*. The left panel is the representative blot and the right panel is the average data. NC, negative scrambled siRNA; GAPDH, α‐tubulin, and Lamin A/C were used as internal controls for total cellular, cytoplasmic, and nuclear protein, respectively. si‐1 and si‐2 are two separated siRNA against *S100A9*. n = 4. Data are mean ± SEM. Two‐tailed paired Student's t‐test (D‐H), or One‐way ANOVA followed by Tukey's post hoc tests (I). *p < 0.05, **p < 0.01, ***p < 0.001.

To investigate the exact role of nuclear S100A9 in hAFs, we transfected hAFs isolated from the TNL amnion with a constructed adenoviral vector carrying a nuclear localization sequence (NLS) to the C‐ and N‐termini of S100A9 (NLS‐S100A9) to specifically express S100A9 in the nucleus. Nuclear overexpression was confirmed with immunofluorescent staining and Western blotting (Figure S2A–C, Supporting Information). However, ELISA assay showed that extracellular S100A9 was not altered by transfection of NLS‐S100A9 in hAFs (Figure S2D, Supporting Information). To screen for pathways affected by nuclear S100A9 in hAFs, transcriptomic sequencing was performed in hAFs with overexpression of S100A9 in the nuclei. Compared to transfection with an empty adenoviral vector, there were 136 down‐regulated and 41 up‐regulated genes (fold change ≥ 1.5 and *p* < 0.05) after nuclear overexpression of S100A9 in hAFs (Figure S2E, Table S1, Supporting Information). The gene set enrichment analysis (GSEA) showed that the down‐regulated gene sets seen with nuclear S100A9 overexpression were associated with cell cycle and DNA replication (**Figure** **3**A; Figure S2F, Supporting Information). Analysis of the differentially expressed genes with the Kyoto Encyclopedia of Genes and Genomes (KEGG) revealed that cell cycle and cellular senescence ranked first and sixth respectively among the enriched KEGG pathways (Figure 3B), which coincides largely with the Gene Ontology (GO) analysis showing that cell division and mitotic cell cycle were the top two enriched GO terms (Figure 3C). The altered genes associated with the cell cycle GO term were illustrated in the heatmap (Figure S2G, Supporting Information), which included *CCNE2* (cyclin E2), *CDK1* (cyclin dependent kinase 1), *E2F1* (E2F transcription factor 1), and *CDKN1A* (cyclin dependent kinase inhibitor 1A, also known as p21). These alterations were subsequently confirmed with qRT‐PCR (Figure 3D). Subsequent Western blotting analysis showed that nuclear overexpression of S100A9 increased the protein abundance of p21 and p53 in hAFs (Figure 3E), which was confirmed by dual immunofluorescent staining showing increased p21 abundance in hAFs with nuclear S100A9 overexpression (Figure S3A, Supporting Information). Consistently, SA‐β‐gal staining revealed an increased ratio of SA‐β‐gal‐positive cells in hAFs with nuclear overexpression of S100A9 (Figure 3F) along with an increased abundance of γH2AX, a sensitive marker of the DNA damage response,^[^ ^13^ ^]^ as demonstrated with immunofluorescence staining (Figure 3G). Moreover, nuclear overexpression of S100A9 increased PGE2 production (Figure 3H) and COX‐2 expression (Figure S3B,C, Supporting Information) in hAFs. By contrast, the expression of COX‐1, a constitutive counterpart of the inducible cyclooxygenase in prostaglandin synthesis, was not affected by nuclear overexpression of S100A9 in hAFs (Figure S3D, Supporting Information). Conversely, small interfering RNA (siRNA)‐mediated knockdown of *S100A9* in hAFs isolated from the TL amnion significantly decreased nuclear S100A9 abundance along with decreased p21 and p53 abundance (Figure 3I). To examine the effect of extracellular S100A9 on cellular senescence in hAFs, hAFs isolated from the TNL amnion were treated with recombinant human S100A9 (rhS100A9; 0.05, 0.5 and 5 µg mL^−1^) for 72 h or with rhS100A9 (0.5 µg mL^−1^) for 24, 48, and 72 h. These time course and concentration‐dependent studies showed that extracellular S100A9 had no effect on the abundance of p53 and p21 protein in hAFs (Figure S3E,F, Supporting Information). These data indicate that nuclear overexpression of S100A9 can trigger cellular senescence in hAFs, whereas extracellular S100A9 had no such effect.

### 2.4 Disruption of Heterochromatin by Nuclear S100A9 in hAFs

**Figure 4:**
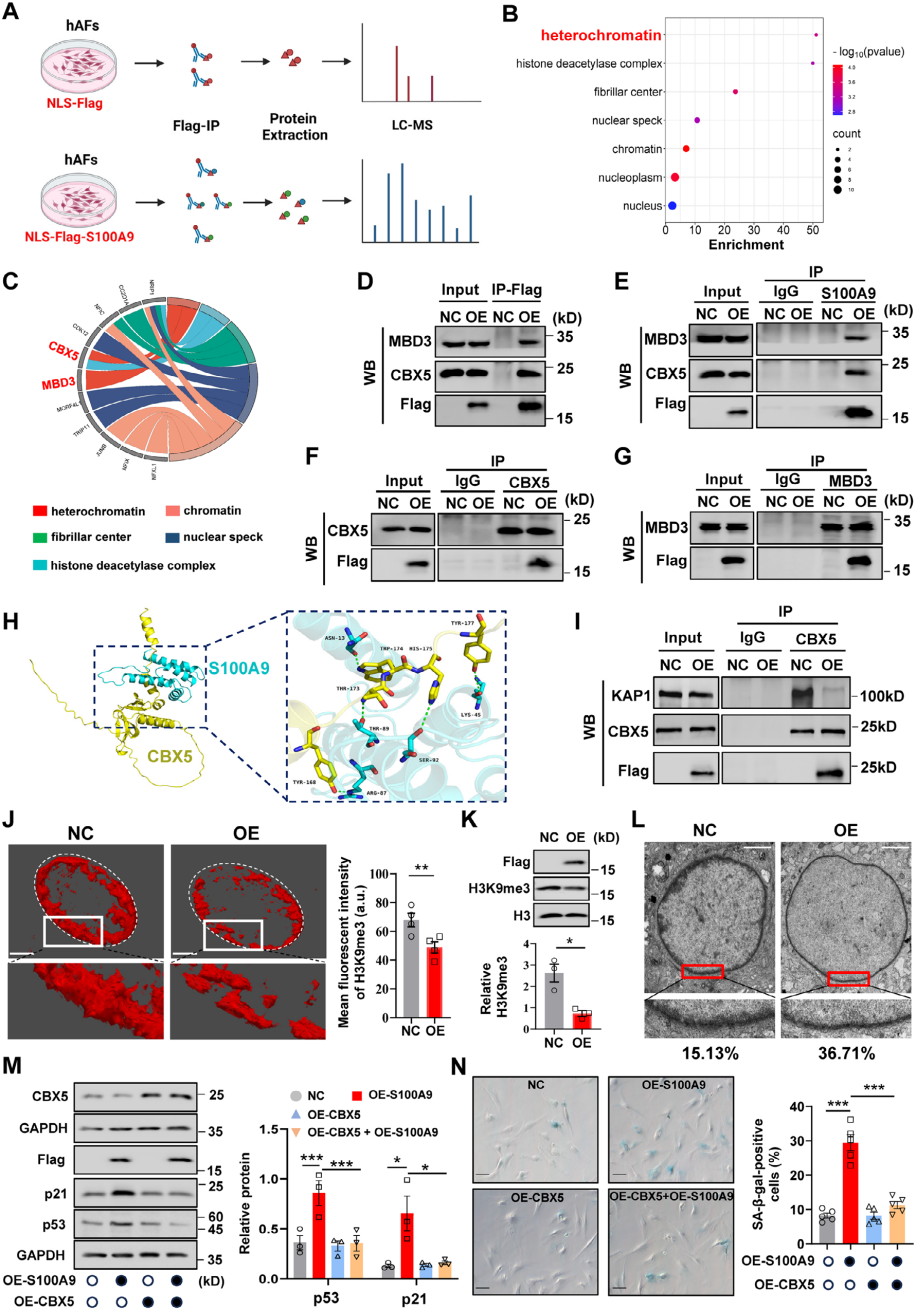
Disruption of heterochromatin by nuclear S100A9 in hAFs. A) Schematic diagram illustrating the workflow of mass spectrometry for identification of S100A9‐interacting proteins in hAFs with or without nuclear S100A9 overexpression. n = 4 per group. B,C) Dot plot (B) and chord plot (C) of the GO cellular component enrichment analysis showing S100A9‐interacting proteins identified with mass spectrometry. D–G) Co‐IP analysis showing CBX5 and MBD3 binding with S100A9 in hAFs with and without nuclear S100A9 overexpression. (D) is immunoprecipitation with anti‐Flag‐conjugated beads, (E) is immunoprecipitation with anti‐S100A9 antibody, (F) is immunoprecipitation with anti‐CBX5 antibody, and (G) is immunoprecipitation with anti‐MBD3 antibody. Input and pre‐immune IgG served as positive and negative controls respectively. n = 3. NC, control with empty vector; OE, nuclear S100A9 overexpression. H) Protein‐protein docking prediction by Z‐DOCK showing the overall co‐complex structure of S100A9 (cyan color) with CBX5 (yellow color). The panel in the large box is the enlarged view of S100A9‐CBX5 binding site. I) Co‐IP analysis showing CBX5 binding with KAP1 in hAFs with and without nuclear S100A9 overexpression. Input and pre‐immune IgG served as positive and negative controls respectively. n = 3. J) 3D reconstruction of z‐stack immunofluorescence images of H3K9me3 (red color) in the nucleus of hAFs with or without nuclear S100A9 overexpression. Scale bars, 2 µm. The mean fluorescence intensity of H3K9me3 (right) was related to Figure S4C. n = 4. K) Western blot analysis showing a decreased abundance of H3K9me3 in hAFs with nuclear S100A9 overexpression as compared to that without nuclear S100A9 overexpression. n = 3. The upper panel is the representative blot and the lower panel is the average data. L) Representative images of transmission electron microscopy (TEM) showing peripheral heterochromatin in hAFs with or without nuclear S100A9 overexpression. The percentages of cells with compromised nuclear peripheral heterochromatin are presented at the bottom of the TEM images in each group. Scale bars, 2 µm. M) Western blot analysis showing the abundance of p53 and p21 in hAFs with or without nuclear S100A9 overexpression in the presence or absence of CBX5 overexpression. n = 3. The left panel is the representative blot and the right panel is the average data. N) SA‐β‐gal staining showing the difference in the percentage of cells positive for SA‐β‐gal (blue color) in hAFs with or without nuclear S100A9 overexpression in the presence or absence of CBX5 overexpression. n = 5. Scale bars, 25 µm. The left panel is the representative image and the right panel is the average data. Data are mean ± SEM. Two‐tailed paired Student's t‐test (J, K), or One‐way ANOVA followed by Tukey's post hoc tests (M, N). *p < 0.05, **p < 0.01, ***p < 0.001.

To screen for potential partners that nuclear S100A9 might interact with in the induction of cellular senescence in hAFs, immunoprecipitation (IP) was performed following transfection of a vector expressing Flag‐tagged S100A9 in the nuclei of hAFs. The proteins precipitated by the anti‐Flag antibody were then subject to analysis with liquid chromatography‐tandem mass spectrometry (LC‐MS) (**Figure** **4**A). A list of candidate transcription factors and cofactors that S100A9 might interact with to modulate heterochromatin were identified in the nucleus, including CBX5, and methyl‐CpG‐binding domain protein 3 (MBD3), a component of the NuRD complex^[^ ^14^ ^]^ (Figure 4B,C; Figure S4A and Table S2, Supporting Information). These interactions between S100A9 and CBX5 or MBD3 were further validated with co‐immunoprecipitation (Co‐IP) assay in hAFs with nuclear S100A9 overexpression (Figure 4D–G). Interestingly, protein‐protein docking prediction with ZDOCK SERVER or HDOCK SERVER showed that the key sequence in CBX5 that might interact with S100A9 ranged from residues 134 to 177 (Docking Score: 1387.324 for ZDOCK and ‐228.52 for HDOCK) (Figure 4H; Figure S4B, Supporting Information), which overlaps with the reported sequence (residues 121–179, C‐terminal chromoshadow domain) that interacts with KRAB‐associated protein 1 (KAP1),^[^ ^15^ ^]^ an essential scaffold for the formation of the repressor complex in heterochromatin formation.^[^ ^16^ ^]^ This overlap indicates that nuclear overexpression of S100A9 may intervene in the interaction between CBX5 and KAP1, resulting in heterochromatin disruption. This notion was supported by further Co‐IP assay showing decreased CBX5 and KAP1 binding upon nuclear overexpression of S100A9 in hAFs (Figure 4I). The disruption of heterochromatin by nuclear S100A9 overexpression was further supported by the finding of decreased H3K9me3 level, a representative hallmark of heterochromatin erosion, in the nuclei of hAFs (Figure 4J–L; Figure S4C,D, Supporting Information). By contrast, overexpression of CBX5 not only counteracted the decrease in H3K9me3 abundance (Figure S4C,D, Supporting Information), but also attenuated the increases in p21 and p53 abundance (Figure 4M; Figure S4E, Supporting Information) and SA‐β‐gal‐positive cells (Figure 4N), which were induced by nuclear S100A9 overexpression in hAFs. Conversely, siRNA‐mediated knockdown of *CBX5* increased the abundance of p21 and p53 (Figure S4F, Supporting Information). These data indicate that nuclear overexpression of S100A9 can induce cellular senescence by disrupting heterochromatin via segregation of the heterochromatin maintenance protein in hAFs.

### 2.5 De‐Repression of LINE1 by Nuclear S100A9 via Heterochromatin Disruption in hAFs

**Figure 5:**
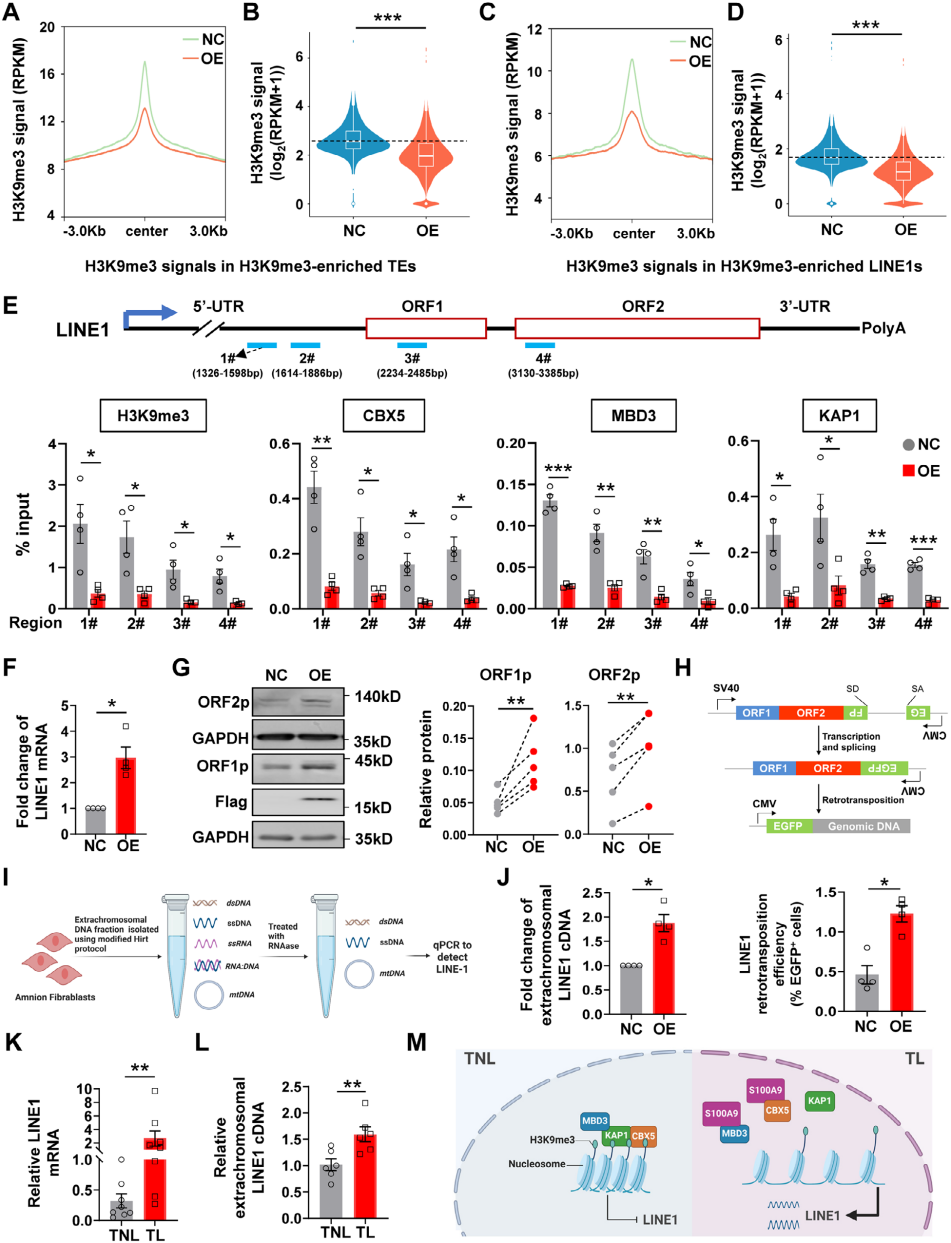
Activation of LINE1 elements by nuclear S100A9 in hAFs. A, B) The metaplot (A) and violin plot (B) show H3K9me3 signals in H3K9me3‐enriched TEs in hAFs with or without nuclear S100A9 overexpression. RPKM, Reads Per Kilobase per Million mapped reads. NC, control with empty vector transfection; OE, nuclear S100A9 overexpression. C, D) The metaplot (C) and violin plot (D) showing H3K9me3 signals in H3K9me3‐enriched LINE1 in hAFs with or without nuclear S100A9 overexpression. E) ChIP‐qRT‐PCR assay showing the enrichment of H3K9me3, CBX5, MBD3, and KAP1 at indicated regions of LINE1 in hAFs with or without nuclear S100A9 overexpression. Pre‐immune IgG served as the negative control, which was shown in Figure S5E (Supporting Information). Top panel showing the regions of the evolutionarily young LINE‐1 family (L1HS), aligned in qRT‐PCR, with the following details: 1# and 2# are aligned to the 5′UTR (1326‐1598 bp and 1614–1886 bp, respectively), 3# is aligned to ORF1 (2234‐2485 bp), and 4# is aligned to ORF2 (3130‐3385 bp). n = 4. F) LINE1 mRNA abundance in hAFs with or without nuclear S100A9 overexpression as measured with qRT‐qPCR with primers aligned to ORF1p. n = 4. Data with primers aligned to ORF2p is shown in Figure S5F (Supporting Information). G) Western blot analysis showing the protein abundance of ORF1p and ORF2p in hAFs with or without nuclear S100A9 overexpression. n = 5. H) Quantification of the *de novo* LINE1 retrotransposition efficiency in hAFs with or without nuclear S100A9 overexpression. n = 4. The upper panel is the schematic diagram showing the structure of LINE1 retrotransposition efficiency reporter pEGFP‐LRE3. I) Schematic diagram showing the procedure of extrachromosomal DNA extraction for qRT‐PCR. J) Extrachromosomal LINE1 cDNA contents in hAFs with or without nuclear S100A9 overexpressing as determined with qRT‐PCR. n = 4. *MT‐ND5*, a mitochondrial gene, was used as the reference gene. K,L) LINE1 mRNA (n = 8 each group, K) and extrachromosomal LINE1 cDNA contents (n = 6 each group, L) in hAFs isolated from TNL and TL groups as determined with qRT‐PCR. TL, term labor; TNL, term no labor. M) Diagram illustrating nuclear S100A9‐mediated heterochromatin erosion in hAFs. Increased S100A9 in the nucleus interacts with heterochromatin proteins CBX5 and MBD3, resulting in their dissociation from heterochromatin, thereby leading to heterochromatin erosion and subsequent de‐repression of LINE1 elements in hAFs at parturition. Data are mean ± SEM. Two‐sided Wilcoxon signed‐rank test (B, D), two‐tailed paired (E‐H, J), or unpaired Student's t‐test (K, L). *p < 0.05, **p < 0.01, ***p < 0.001.

Given that constitutive heterochromatin is crucial for silencing TEs across diverse evolutionarily distant organisms,^[^ ^7e,f^ ^]^ Cleavage Under Targets and Tagmentation (CUT&Tag) analysis using an H3K9me3 antibody was conducted to examine whether the enrichment of H3K9me3 at TE regions was altered by nuclear overexpression of S100A9 in hAFs, followed by high‐throughput sequencing. As expected, nuclear overexpression of S100A9 in hAFs significantly reduced the overall enrichments of H3K9me3 at whole genome regions (Figure S5A, Supporting Information), especially at TE regions, including LINE1, SINE (short interspersed nuclear elements) and LTR (long terminal repeats) elements (**Figure** **5**A–D; Figure S5B–D, Supporting Information). Since LINE1 are the most abundant autonomous retrotransposons that are normally silenced in primates,^[^ ^7b,f^ ^]^ and increased LINE1 retrotransposition is associated with cellular senescence in a variety of non‐gestational tissues,^[^ ^8^ ^]^ we subsequently investigated whether nuclear S100A9 overexpression de‐repressed LINE1 via segregation of the heterochromatin maintenance protein from LINE1 elements to promote cellular senescence in hAFs with chromatin immunoprecipitation (ChIP) assay. This showed that the enrichment of heterochromatin maintenance proteins including CBX5, MBD3 and KAP1 at LINE1 elements was all significantly decreased along with decreased H3K9me3 enrichment upon nuclear S100A9 overexpression in hAFs (Figure 5E; Figure S5E, Supporting Information). Consistently, qRT‐PCR and Western blotting showed that LINE1 transcripts (Figure 5F; Figure S5F, Supporting Information) and the coded proteins ORF1p and ORF2p (Figure 5G) were markedly up‐regulated by nuclear S100A9 overexpression in hAFs. Because aberrant LINE1 expression is linked to altered retrotransposon activity,^[^ ^8b–d^ ^]^ we measured the *de novo* LINE1 retrotransposon efficiency in hAFs by using a well‐characterized EGFP‐based reporter system (Figure 5H).^[^ ^17^ ^]^ As expected, a higher LINE1 retrotransposon activity was detected in hAFs with nuclear S100A9 overexpression (Figure 5H) along with increased LINE1 cDNA in total and extrachromosomal DNA contents (Figure 5I,J; Figure S5G, Supporting Information). Conversely, siRNA‐mediated knockdown of *S100A9* reduced LINE1 mRNA and cDNA contents significantly in hAFs isolated from the TL amnion (Figure S5H‐J, Supporting Information). Moreover, LINE1 mRNA, total and extrachromosomal LINE1 cDNA contents were also significantly increased in hAFs isolated from the TL amnion as compared to the TNL amnion (Figure 5K,L; Figure S5K, Supporting Information). In addition to LINE1, we also examined the effect of nuclear overexpression of S100A9 on human endogenous retroviruses (HERV)‐K, an evolutionarily young subfamily of HERVs that has been reported to be associated with aging.^[^ ^18^ ^]^ The result showed that HERV‐K expression was not affected by nuclear overexpression of S100A9 in hAFs (Figure S5L, Supporting Information). Taken together, these results revealed a novel role of nuclear S100A9 in the relief of LINE1 repression through disruption of heterochromatin associated with LINE1 elements via segregation of the heterochromatin maintenance protein in hAFs (Figure 5M).

### 2.6 De‐Repression of LINE1 Causes Cellular Senescence Via Elicitation of the Type I IFN Response in hAFs with Nuclear S100A9 Overexpression

**Figure 6:**
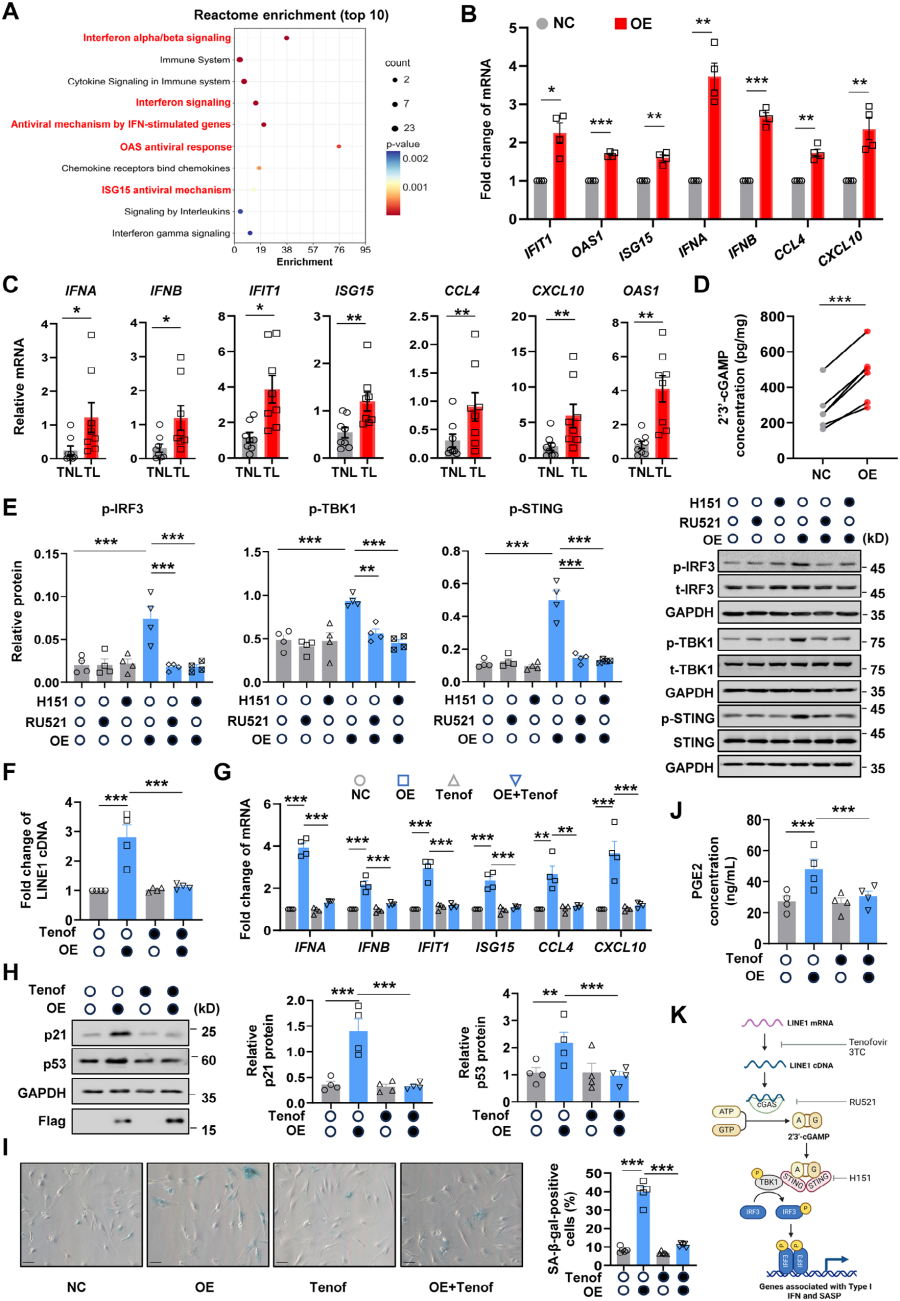
Activation of LINE1 by nuclear S100A9 induces type I IFN response and cellular senescence in hAFs. A) Reactome pathway enrichment analysis showing the pathway enrichment of up‐regulated expressed genes as revealed with transcriptomic sequencing in hAFs with and without nuclear S100A9 overexpression (n = 3 per group). B) qRT‐PCR analysis showing the abundance of mRNA associated with type I IFN responses and SASP in hAFs with or without nuclear S100A9 overexpression. n = 4. NC, control with empty vector transfection; OE, nuclear S100A9 overexpression. C) qRT‐PCR analysis showing the abundance of mRNA associated with type I IFN response and SASP in hAFs isolated from TL (n = 8) and TNL (n = 8). TL, term labor; TNL, term no labor. D) Measurement with ELISA showing the difference of 2′3’‐cGAMP concentration in hAFs with or without nuclear S100A9 overexpression. n = 5. E) Western blot analysis showing the difference in protein abundance of p‐STING, p‐IRF3, and p‐TBK1 in hAFs with or without nuclear S100A9 overexpression in the presence or absence of cGAS inhibitor RU521 and STING inhibitor H151. n = 4. The left panel is the average data and the right panel is the representative blot. F, G) The difference of LINE1 cDNA contents (F) and abundance of mRNA associated with type I IFN response and SASP (G) in hAFs with or without nuclear S100A9 overexpression in the presence or absence of Tenofovir (Tenof). n = 4. H) Western blot analysis showing the difference in protein abundance of p21 and p53 in hAFs with or without nuclear S100A9 overexpression in the presence or absence of Tenof. n = 4. Left panel is the representative blot and the right panel is the average data. I) SA‐β‐gal staining of hAFs with or without nuclear S100A9 overexpression in the presence or absence of Tenof. n = 5. The Left panel is the representative image and the right panel is the average data. Scale bars, 25 µm. J) ELISA showing PGE2 production in hAFs with or without nuclear S100A9 overexpression in the presence or absence of Tenof. n = 4. K) Schematic diagram showing the activation of cGAS‐STING pathway, type I IFN response, and SASP by LINE1 in hAFs with nuclear S100A9 overexpression. Data are mean ± SEM. Two‐tailed paired (B, D) or unpaired Student's t‐test (C), or One‐way ANOVA followed by Tukey's post hoc tests (E‐J). *p < 0.05, **p < 0.01, ***p < 0.001.

Accumulating evidence indicates that intermediates of activated LINE1 transposition, i.e., cytosolic LINE1 cDNA, can be recognized by cGAS to activate the cGAS‐STING pathway, which can subsequently elicit the type I IFN response with consequent cellular senescence in non‐gestational tissues.^[^ ^8^ ^]^ In support of this, GSEA analysis of the transcriptomic sequencing data obtained from hAFs showed that the type I IFN response (i.e., IFN α/β signaling) was indeed activated by nuclear S100A9 overexpression (Figure S6A, Supporting Information). Reactome enrichment analysis of the up‐regulated genes by nuclear S100A9 overexpression also revealed that many of the top ten enriched pathways were related to the type I IFN response (**Figure** **6**A). Correspondingly, the expression of genes associated with the type I IFN response and SASP, including *IFNA*, *IFNB*, *IFIT1* (interferon‐induced protein with tetratricopeptide repeats 1 and 3), *OAS1* (2′‐5′‐Oligoadenylate synthetase 1), *ISG15* (interferon‐stimulated gene 15), *CCL4* (C‐C motif chemokine ligand 4), *CXCL10* (C‐X‐C motif chemokine ligand 10), etc., were all significantly upregulated by nuclear S100A9 overexpression in hAFs (Figure 6B; Figure S6B, Supporting Information), as well as in hAFs isolated from the TL amnion as compared to the TNL amnion (Figure 6C). In line with the activation of cGAS‐STING pathway following LINE1 de‐repression,^[^ ^8^ ^]^ the concentration of 2′3′‐cGAMP (2′3′‐cyclic GMP‐AMP), a cGAS downstream nucleotide messenger,^[^ ^19^ ^]^ was significantly increased by nuclear S100A9 overexpression in hAFs (Figure 6D), along with increased phosphorylation of STING, TANK‐binding kinase 1 (TBK1) and IFN regulatory factor 3 (IRF3), the three molecules downstream of the cGAS‐STING pathway^[^ ^19^ ^]^ (Figure 6E). Conversely, inhibition of cGAS and STING activities with RU521 and H151 respectively blocked not only the increases in the phosphorylation of STING, TBK1 and IRF3 (Figure 6E), but also the increases in transcripts associated with the type I IFN response and SASP, and protein abundance of p21 and p53 (Figure S6C,D, Supporting Information) induced by nuclear S100A9 overexpression in hAFs. This indicates that the cGAS‐STING signaling pathway is involved in the induction of cellular senescence by nuclear overexpression of S100A9 in hAFs. Moreover, Tenofovir and Lamivudine (3TC), the two nucleoside analog reverse‐transcriptase inhibitors (NRTIs) that have been used to inhibit LINE1 reverse transcription,^[^ ^20^ ^]^ effectively attenuated LINE1 retrotransposition (Figure 6F; Figure S6E, Supporting Information), type I IFN response and SASP (Figure 6G; Figure S6F, Supporting Information) induced by nuclear overexpression of S100A9 in hAFs along with decreased p21 and p53 abundance (Figure 6H; Figure S6G, Supporting Information), reduced proportion of SA‐β‐gal‐positive cells (Figure 6I), and decreased COX‐2 expression and PGE2 production (Figure 6J; Figure S6H, Supporting Information). In contrast, Etravirine, a non‐nucleoside analog reverse‐transcriptase inhibitor (NNRTI) used in anti‐human immunodeficiency virus (HIV) therapy with no effect on LINE1 reverse transcription,^[^ ^20c^ ^]^ failed to alleviate the type I IFN response and cellular senescence‐induced by nuclear overexpression of S100A9 in hAFs (Figure S6I‐K, Supporting Information). These data substantiate that nuclear overexpression of S100A9 promotes cellular senescence and intensifies SASP and PGE2 production via elicitation of the type I IFN response following LINE1 de‐repression in hAFs (Figure 6K).

### 2.7 De‐Phosphorylation of S100A9 at Thr 113 is Required for Nuclear Translocation in hAFs

**Figure 7:**
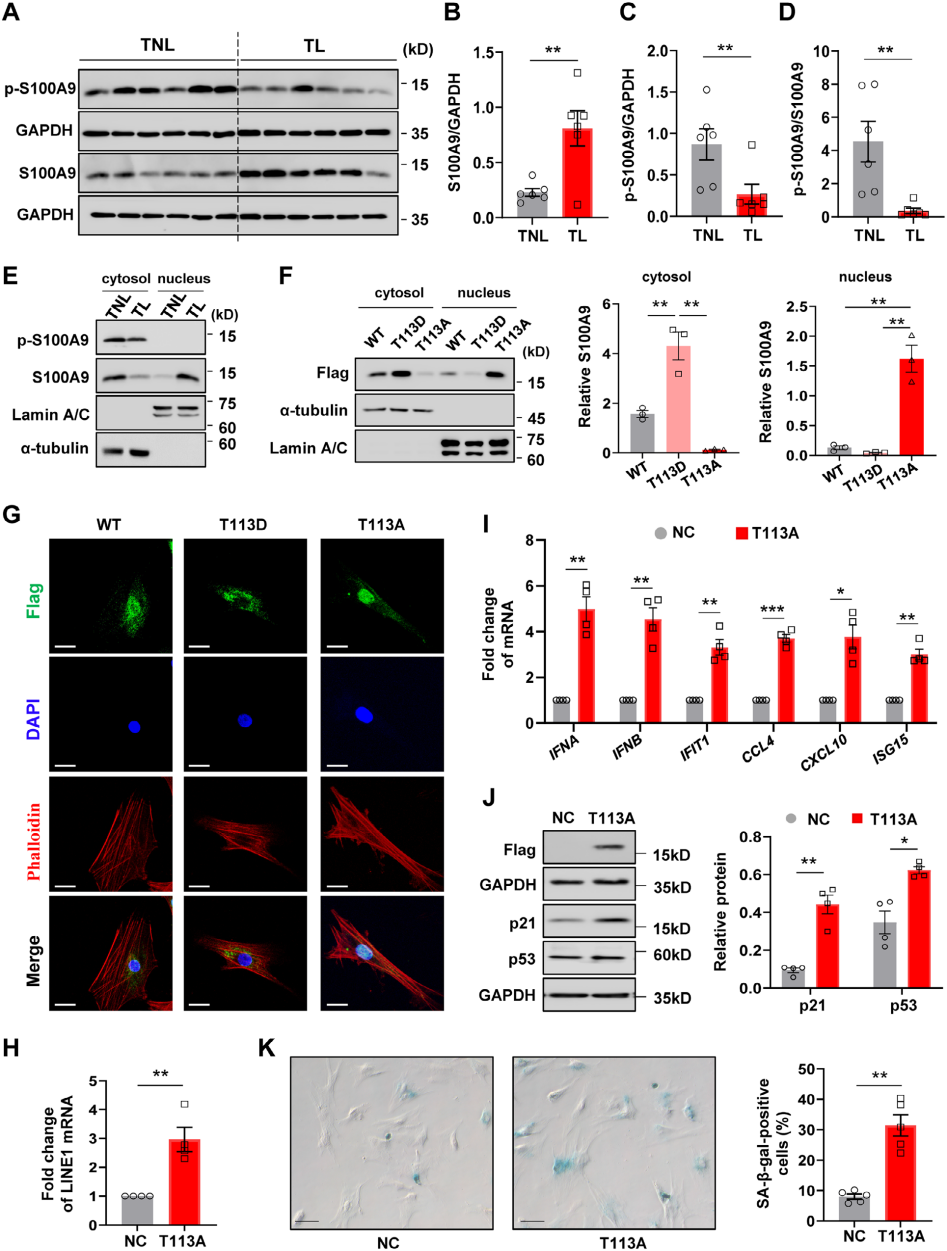
De‐phosphorylation of S100A9 at Thr 113 enables its nuclear translocation in hAFs. A–D) Western blotting analysis showing the differences of total S100A9 and phosphorylated S100A9 at Thr 113 (p‐S100A9) in hAFs isolated from TL (n = 6) and TNL (n = 6). (A) is the Western blot and (B–D) is the average data. TL, term labor; TNL, term no labor. E) Western blot analysis showing the abundance of S100A9 and p‐S100A9 in the cytosolic and nuclear fractions of hAFs isolated from TL and TNL. α‐tubulin and Lamin A/C were used as internal controls for cytoplasmic and nuclear protein, respectively. n = 3. F and G) Western blot analysis (F) and immunofluorescent staining (G) of Flag (green color) showing the difference of cytosolic and nuclear distributions of wild‐type S100A9, T113D mutant S100A9 and T113A mutant S100A9 in hAFs. The left panel of (F) is the representative blot, and the right panel of (F) is the average data. n = 3. Nuclei and cytoskeleton were counterstained with DAPI (blue color) and phalloidin (red color) respectively. Scar bar, 25 µm. H and I) Measurement with qRT‐qPCR showing the difference of LINE1 transcripts (H), and the mRNA associated with type I IFN response and SASP (I) in hAFs with or without overexpression of S100A9 carrying T113A mutant. n = 4. J) Western blot analysis showing the difference of p53 and p21 protein in hAFs with or without overexpression of S100A9 carrying T113A mutant. n = 4. The left panel is the representative blot and the right panel is the average data. K) SA‐β‐gal (blue) staining of hAFs with or without overexpression of S100A9 carrying T113A mutant. Scale bars, 25 µm. n = 5. The left panel is the representative image and the right panel is the average data. Data are mean ± SEM. Mann–Whitney U test (C), Two‐tailed unpaired (B, D) or paired Student's t‐test (H‐K), or One‐way ANOVA followed by Tukey's post hoc tests (F). *p < 0.05, **p < 0.01, ***p < 0.001.

Given the crucial role of the nuclear S100A9 in the induction of cellular senescence in hAFs, we next explored the molecular mechanism underlying S100A9 nuclear translocation in hAFs. Interestingly, we found a significant reduction in S100A9 phosphorylation at Thr 113 (p‐S100A9) in hAFs isolated from the TL amnion as compared to that from the TNL amnion (**Figure** **7**A–D). Moreover, the abundance of p‐S100A9 correlated negatively with the abundance of S100A9 in the nucleus and the levels of p21 or p53 in hAFs (Figure S7A‐C, Supporting Information). Furthermore, p‐S100A9 was found to be exclusively localized in the cytosol but not in the nuclei of hAFs (Figure 7E; Figure S7D, Supporting Information), indicating that de‐phosphorylation of S100A9 at Thr 113 is required for its nuclear translocation. To confirm this, vectors carrying Flag‐tagged wild‐type, T113A (Thr→Ala) de‐phosphorylation‐mimic mutant, or T113D (Thr→Asp) phosphorylation‐mimic mutant S100A9 were constructed and transfected into hAFs. Immunofluorescent staining and Western blotting analyses showed that T113D phosphorylation‐mimic mutant S100A9 was mainly localized in the cytosol (Figure 7F,G), while T113A de‐phosphorylation‐mimic mutant S100A9 had higher nuclear accumulation than wild‐type S100A9 or T113D phosphorylation‐mimic mutant S100A9 (Figure 7F). Accordingly, overexpression of T113A de‐phosphorylation‐mimic mutant S100A9 led to LINE1 de‐repression (Figure 7H), elicitation of the type I IFN responses, and consequently cellular senescence in hAFs (Figure 7I–K), while overexpression of T113D phosphorylation‐mimic mutant S100A9 had no such effects (Figure S7E,F, Supporting Information). Moreover, Co‐IP assay showed that there was abundant binding between CBX5 and KAP1, but almost no binding of CBX5 and S100A9 in hAFs with T113D phosphorylation‐mimic mutant S100A9 overexpression (Figure S7G,H, Supporting Information). These results indicate that de‐phosphorylation of S100A9 at Thr 113 is required for its nuclear translocation, where it de‐represses LINE1 via segregation of the heterochromatin maintenance protein, which further induces cellular senescence in hAFs.

### 2.8 Induction of Preterm Birth by Nuclear S100A9 in the Mouse

**Figure 8:**
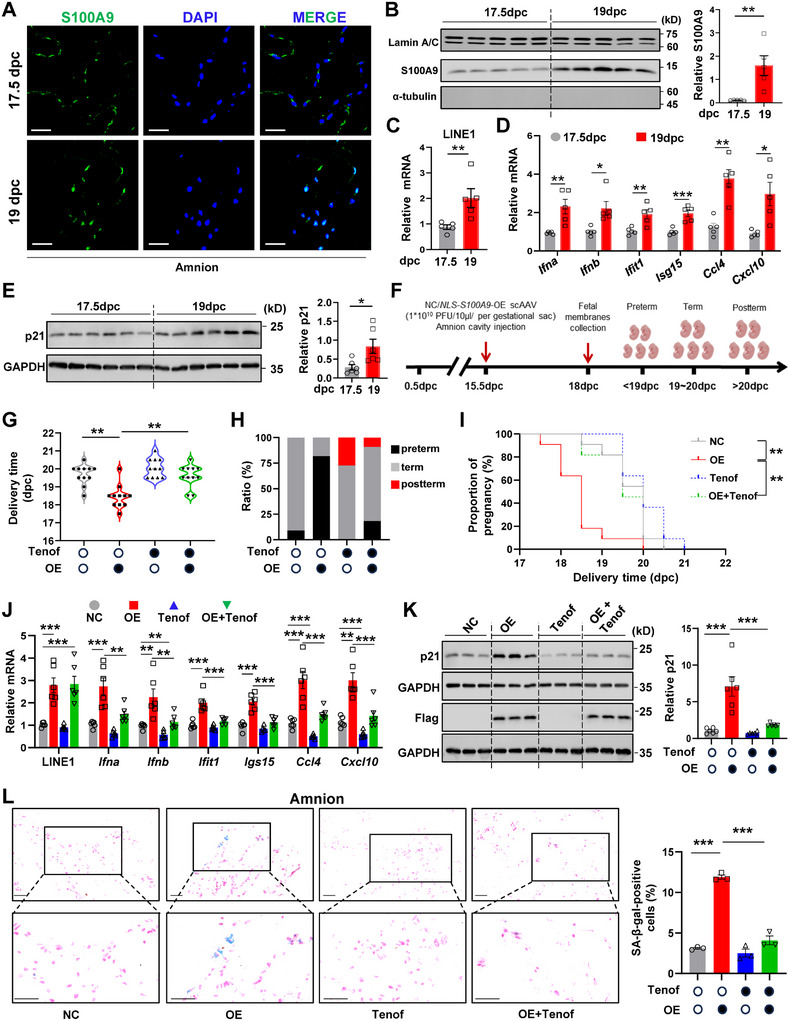
Nuclear overexpression of S100A9 in the fetal membranes causes preterm birth in the mouse. A) Representative images of immunofluorescence staining showing intense S100A9 staining (green color) in the cell nuclei of the mouse amnion layer at 19 dpc. Nuclei were counterstained with DAPI (blue color). Scale bars, 25 µm. n = 3. B) Western blot analysis showing increased abundance of S100A9 in the nuclear fraction extracted from the mouse fetal membranes at 19 dpc as compared to 17.5 dpc. n = 5 per group. The left panel is the blot and the right panel is the average data. C,D) Measurement with qRT‐PCR showing increased abundance of LINE1 (C), mRNA associated with type I IFN response, and SASP (D) in the mouse fetal membranes at 19 dpc as compared to 17.5 dpc. n = 5 per group. E) Western blot analysis showing increased p21 protein abundance in the mouse fetal membranes at 19 dpc as compared to 17.5 dpc. n = 6 per group. The left panel is the blot and the right panel is the average data. F) Time‐line illustrating the procedure of S100A9 administration in pregnant mice. G,H) Effects of intra‐amniotic injection of scAAV vector expressing S100A9 (1 × 10^10^ TU/10 µl/ per gestational sac at 15.5 dpc) in the nucleus in the presence or absence of Tenofovir (Tenof; 2 nmol/10 µl/per gestational sac) on delivery time (G) and rates of preterm and post‐term birth (H). n = 11 per group. NC, control empty vector. OE, scAAV vector expressing S100A9 in the nucleus. I) Gestational lengths in mice with an intra‐amniotic injection of scAAV vector expressing S100A9 in the nucleus in the presence or absence of Tenof (2 nmol/10 µl/per gestational sac). n = 11 per group. J) Measurement with qRT‐PCR showing the abundance of LINE1 transcripts and mRNA associated with type I IFN response and SASP in the mouse fetal membranes with intra‐amniotic injection of scAAV vector expressing S100A9 in the nucleus in the presence or absence of Tenof (2 nmol/10 µl/per gestational sac). n = 6 per group. K) Western blot analysis showing the protein abundance of p21 in the mouse fetal membranes with an intra‐amniotic injection of scAAV vector expressing S100A9 in the nucleus in the presence or absence of Tenof (2 nmol/10 µl/per gestational sac). n = 6 per group. The left panel is the representative blot and the right panel is the average data. L) SA‐β‐gal (blue color) staining of the amnion layer of the mouse fetal membranes with an intra‐amniotic injection of scAAV vector expressing S100A9 in the nucleus in the presence or absence of Tenof (2 nmol/10 µl/per gestational sac). Nuclei were counterstained with nuclear fast red (red color). n = 3 per group. Scale bars, 50 µm. The Left panel is the representative image and the right panel is the average data. Data are mean ± SEM. Two‐tailed unpaired Student's t‐test (B‐E), Gehan‐Breslow‐Wilcoxon test (I), or One‐way ANOVA followed by Tukey's post hoc tests (G, J‐L). *p < 0.05, **p < 0.01, ***p < 0.001.

The role of nuclear S100A9 in parturition was further investigated in pregnant mice. The mouse fetal membranes are comprised of the amnion and yolk sac membranes.^[^ ^21^ ^]^ Immunofluorescent staining showed an intense staining of S100A9 in the nuclei in both the amnion and yolk sac membranes at 19 days post‐coitum (dpc) (**Figure** **8**A; Figure S8A, Supporting Information). Consistently, Western blotting analysis showed that the abundance of nuclear S100A9 was significantly higher at 19 dpc than at 17.5 dpc (Figure 8B). Correspondingly, transcripts of LINE1 (Figure 8C) and those related to the type 1 IFN response and SASP (*Ifna, Ifnb, Isg15, Ifit1, Ccl4, Cxcl10*) (Figure 8D) were significantly increased along with increased p21 protein abundance (Figure 8E) in the fetal membranes at 19 dpc as compared to 17.5 dpc. These data suggest that nuclear translocation of S100A9, LINE1 de‐repression, and cellular senescence are increased in the mouse fetal membranes toward the end of gestation.

Intra‐amniotic injection of the self‐complementary adeno‐associated virus (scAAV) vector (1 × 10^10^ transducing units (TU)/10 µL/per gestational sac) expressing S100A9 specifically in the nucleus at 15.5 dpc (Figure 8F; Figure S8B,C, Supporting Information) had no effect on fetal mortality (Figure S8D), but led to a 81.8% preterm birth rate (Figure 8G–I), along with increased transcripts of LINE1 and those associated with the type I IFN response and SASP (Figure 8J) in the mouse fetal membranes. Meanwhile, the abundance of p21 protein (Figure 8K), and the proportion of p21‐ and SA‐β‐gal‐positive cells (Figure 8L; Figurse S8E and S9A‐C, Supporting Information) were also significantly increased in the mouse fetal membranes. Notably, intra‐amniotic injection of the reverse transcription inhibitor Tenofovir (0.2 nmol/10 µL/per gestational sac) not only tended to prolong gestational age by itself, but also delayed preterm birth induced by intra‐amniotic injection of scAAV vector expressing S100A9 in the nucleus (Figure 8G–I), along with decreased transcripts of genes associated with the type I IFN response and SASP (Figure 8J), p21 protein (Figure 8K), and the proportion of cells positive for p21‐ and SA‐β‐gal (Figure 8L; Figures S8E and S9A–C, Supporting Information) in the mouse fetal membranes. Moreover, intra‐amniotic injection of scAAV vector expressing S100A9 in the nucleus also significantly increased PGE2 levels in the mouse fetal membranes, an effect also attenuated by Tenofovir as well (Figure S8F, Supporting Information).

## 3 Discussion

There are three major cell types in the human fetal membranes, i.e., amnion epithelial cells, chorion trophoblasts, and mesenchymal fibroblasts.^[^ ^11a,c^ ^]^ A series of studies have identified cellular senescence in amnion epithelial cells and chorionic trophoblasts.^[^ ^2b–d^ ^]^ However, it is not known whether mesenchymal fibroblasts of the fetal membranes, an important source of ECM, pro‐inflammatory and pro‐labor factors, also undergo cellular senescence toward the end of gestation to participate in parturition. In this study, we found that cellular senescence in hAFs was an important feature of parturition as well. We demonstrated that nuclear translocation of S100A9 triggered cellular senescence in hAFs through segregation of the heterochromatin maintenance protein with resultant heterochromatin erosion and LINE1 de‐repression. De‐repression of LINE1 further activated the cGAS‐STING pathway and elicitation of the type I IFN response, which ultimately led to cellular senescence in hAFs. Of interest, increased nuclear translocation of S100A9 was observed only in hAFs but not in hEPCs despite cellular senescence occurring in both cell types at parturition, suggesting differential mechanisms may underlie cellular senescence in these two cell types. Previous studies have shown that telomere erosion and oxidative stress may underlie cellular senescence in hEPCs.^[^ ^2b–d,f^ ^]^ In the present study, we demonstrated that nucleus‐translocated S100A9 may be a cause of cellular senescence in hAFs. As hAFs are an important source of ECM components and pro‐inflammatory factors of the fetal membranes, the induction of cellular senescence by nucleus‐translocated S100A9 in hAFs may be crucial to ECM remodeling and inflammatory reactions required for membrane rupture and labor onset. This crucial role of nucleus‐translocated S100A9 in parturition was illustrated by induction of preterm birth following intra‐amniotic injection of scAAV vector expressing S100A9 in the nucleus in the mouse.

It is known that S100A9 is predominantly expressed in myeloid cells, such as neutrophils and monocytes, and functions primarily as a secretory chemoattractant or pro‐inflammatory cytokine that interacts with the toll‐like receptor (TLR), receptor for advanced glycation end products or CD68, either on its own or by forming heterodimers or heterotetramers with S100A8.^[^ ^9^, ^22^ ^]^ Interestingly, there is one study reporting that S100A9 may induce bone marrow stromal cell senescence as an extracellular cytokine via activation of the TLR4 receptor.^[^ ^23^ ^]^ However, in our study, we found that nuclear rather than extracellular S100A9 took part in the induction of cellular senescence in hAFs. We do not currently have a plausible explanation for this discrepancy. More studies are required to confirm the effect of extracellular S100A9 on cellular senescence in different cell types. In line with our findings, there are two studies reporting the presence of S100A9 in the nuclei of other cell types.^[^ ^24^ ^]^ Schonthaler et al. have found that S100A9 is localized in the nuclei of keratinocytes where it modulates the expression of complement factor C3 via chromatin binding and remodeling at a region upstream to the *C3* transcription start site rather than as a transcription factor.^[^ ^24a^ ^]^ Song et al. have demonstrated that S100A9 in the nuclei of MCF‐7 breast cancer cells may function as a coactivator for transcription factors, such as c‐Fos, CCAAT/enhancer‐binding protein β, Signal transducer and activator of transcription 3, etc.,.^[^ ^24b^ ^]^ To the best of our knowledge, the present study is the first to show that S100A9 translocated to the nucleus may function as a heterochromatin disruptor via segregation of the heterochromatin maintenance protein CBX5 from the interaction with other heterochromatin maintenance proteins such as KAP1. Of interest, the predicted CBX5 sequence responsible for binding with S100A9 overlaps with the reported sequence which binds with KAP1,^[^ ^15^ ^]^ suggesting that nuclear overexpression of S100A9 may compete with KAP1 for binding with CBX5, resulting in the loss of CBX5 from the heterochromatin maintenance protein complex. It has been reported CBX5 usually serves as an adapter to recruit effector molecules to heterochromatin, including H3K9 methyltransferases (SUV39H1, SETDB1, G9a, etc.) for methylation of H3K9.^[^ ^25^ ^]^ Alternatively, CBX5 may also enhance H3K9 methylation by stabilizing H3K9 methyltransferases or preventing H3K9 de‐methylation.^[^ ^26^ ^]^ Thus, the loss of CBX5 from the heterochromatin maintenance complex may decrease the enrichment of H3K9me3 resulting in the disruption of heterochromatin integrity, as has been demonstrated in several previous studies.^[^ ^8c,d^ ^]^ However, it is uncertain whether methyltransferases or demethylases involved in H3K9 methylation may be affected directly by nuclear S100A9 in hAFs.

Previous studies have shown that phosphorylation of S100A9 at Thr 113 not only contributes to microtubule reorganization in monocytes,^[^ ^27^ ^]^ but also facilitates S100A9 secretion into the extracellular space in neutrophils.^[^ ^28^ ^]^ Here, we identified that de‐phosphorylation of S100A9 at Thr 113 enabled its nuclear translocation. Similarly, de‐phosphorylation has also been shown to mediate the nuclear import of a variety of other proteins, including nuclear factor of activated T cells, nuclear xenobiotic receptor CAR2, Y‐box‐binding protein 1, cofilin, etc.^[^ ^29^ ^]^ Activation of p38 mitogen‐activated protein kinase is reported to be responsible for S100A9 phosphorylation at Thr 113 in monocytes and neutrophils.^[^ ^27^, ^30^ ^]^ However, the factor driving de‐phosphorylation of S100A9 in hAFs in parturition remains to be determined.

Emerging evidence indicates that genomic instability is a prominent contributor to cellular senescence, and condensed heterochromatin is required for safeguarding genomic integrity, at least in part, through LINE1 silencing.^[^ ^5^, ^31^ ^]^ The expression and retrotransposition of LINE1 elements have been reported to be associated with a number of physiological events, including zygotic genome activation, early embryonic development, neurological disorders, and cancers.^[^ ^32^ ^]^ A series of studies have demonstrated that LINE1 is de‐repressed and aberrantly transcribed to induce cellular senescence via activation of the cGAS‐STING‐type I IFN response in non‐gestational tissues.^[^ ^8^ ^]^ It is known that the cGAS‐STING pathway is primarily activated by the presence of cytosolic DNA, which may derive from damaged DNA, retrotransposons, or viral infections. Upon detection of cytosolic DNA, cGAS synthesizes cGAMP, which binds and activates STING. Activated STING further induces the production of type I IFNs and other inflammatory cytokines, which are part of the innate immune response,^[^ ^33^ ^]^ and are well‐recognized to play a crucial role in the initiation and maintenance of cellular senescence.^[^ ^34^ ^]^ Our study also revealed that the induction of cellular senescence by nuclear overexpression of S100A9 was accompanied by activation of the cGAS‐STING pathway and the type I IFN response in hAFs. Moreover, we also found that COX‐2 expression and PGE2 production were significantly increased by nuclear overexpression of S100A9, which could be blocked by a reverse transcription inhibitor in hAFs, indicating that the induction of COX‐2 expression and PGE2 production is a consequence of LINE1 retrotransposition‐sponsored cellular senescence in hAFs. Given that senescent cells can release a variety of SASP factors, including pro‐inflammatory cytokines that are well‐recognized inducers of COX‐2 expression and PGE2 production,^[^ ^35^ ^]^ we believe that the induction of COX‐2 expression and PGE2 production by nuclear S100A9 is attributed to SASP factors released from senescent cells.

Notably, apart from LINE1, other TEs, such as the recently integrated HERV‐K subgroup, were also reported to be involved in aging.^[^ ^18^ ^]^ Autio, et al.^[^ ^18b^ ^]^ found a slight elevation in the expression levels of HERV‐K in peripheral blood mononuclear cells in older individuals compared to younger counterparts. A recent elegant study from Liu's lab reported that the activation of endogenous retroviruses such as HERV‐K could also activate innate immunity and trigger cellular senescence in human mesenchymal progenitor cells.^[^ ^18c^ ^]^ However, HERV‐K was not affected by nuclear S100A9 in our cell system. Whether other repetitive elements are also involved in the induction of cellular senescence by nuclear overexpression of S100A9 in hAFs awaits further studies.

NRTIs are the most widely used drugs for HIV treatment. A series of studies have demonstrated that NRTIs, such as Tenofovir and 3TC, can mitigate the type I IFN and SASP responses and counteract cellular senescence triggered by LINE1 cDNA accumulation in multiple cell types and animals.^[^ ^8b–e^ ^]^ Here we found that Tenofovir and 3TC were not only capable of ameliorating the aging phenotype of hAFs but also delayed the time of delivery in pregnant mice with nuclear overexpression of S100A9 in the mouse fetal membranes. Considering that Tenofovir use in pregnancy has generally been thought to be safe for both mother and fetus,^[^ ^36^ ^]^ there may be potential in developing Tenofovir as a therapeutic drug for the prevention of preterm birth. In addition to the inhibition of de‐repression of LINE1 with Tenofovir, the molecular mechanism identified that regulates nuclear translocation of S100A9 may also serve as a therapeutic target for the prevention of preterm birth.

## 4 Conclusion

We have discovered a novel role for nuclear‐translocated S100A9 as a heterochromatin disruptor to de‐repress LINE1, which subsequently activates the cGAS‐STING pathway resulting in the activation of the type I IFN response and consequent senescence of hAFs at parturition. We have also demonstrated that de‐phosphorylation of S100A9 at Thr 113 is required for its nuclear translocation. Our findings not only expand knowledge on the function of nuclear‐translocated S100A9, but also provide new insights into the mechanism underlying cellular senescence in hAFs of the fetal membranes at parturition.

## 5 Experimental Section

### Collection of Human Fetal Membranes and Processing of the Amnion Tissue

This study was approved by the Ethics Committee of Ren Ji Hospital, Shanghai Jiao Tong University School of Medicine (approval code: KY2023‐007‐B). All ethical regulations relevant to participants of human research were followed. Human fetal membranes were collected from uncomplicated pregnancies at delivery with written informed consent. Participating subjects were divided into four groups: elective cesarean section without labor at term (designated as term no labor, TNL); spontaneous labor at term (designated as term labor, TL); preterm elective cesarean section without labor for maternal or fetal conditions including placenta previa, vasa previa, and fetal distress (designated as preterm no labor, PNL); and spontaneous preterm labor with no indication of infection (designated as preterm labor, PL). Pregnancies with complications such as preeclampsia, fetal growth restriction, gestational diabetes, and chorioamnionitis were excluded from this study. The demographic features of recruited women are listed in Tables S3 and S4, (Supporting Information).

The amnion layer was collected by peeling it off the fetal membranes immediately after delivery for processing as follows. Several amnion tissue pieces were snap‐frozen in liquid nitrogen, and then sectioned for SA‐β‐gal staining, or processed for extraction of total RNA extraction, and total and nuclear protein for subsequent analysis with qRT‐PCR and Western blotting respectively. Parts of the amnion tissue were rolled prior to fixation in 10% formalin and then embedded in paraffin wax for subsequent sectioning and immunohistochemical or immunofluorescence staining. For isolation of primary hAFs, the entire amnion layer from the reflected membranes was used and processed as follows.

### Isolation of Primary hAFs

The amnion tissue was washed with sterile cold normal saline containing antibiotics (Life Technologies Inc.; #15640055) to remove residual blood, and then subject to consecutive digestions with 0.125% trypsin (Life Technologies Inc.; #9002‐07‐7) and 0.1% collagenase (Sigma–Aldrich; #C0130) to isolate hAFs following a protocol described previously.^[^ ^37^ ^]^ The identity of cells was verified by immunofluorescent staining for the mesenchymal cell marker vimentin and the epithelial marker cytokeratin‐7. More than 95% of the cells were vimentin‐positive (Figure S1E, Supporting Information), which was consistent with the previous report.^[^ ^37^ ^]^

To compare the abundance of S100A9, p‐S00A9, COX‐2, p21, and p53 protein in hAFs isolated from the TNL and TL groups, nuclear protein or total protein was extracted from isolated hAFs for analysis with Western blotting. To compare the LINE1 transcripts, LINE1 cDNA and those transcripts associated with the type I IFN response and SASP in hAFs between the TL and TNL groups, total RNA, cellular and extrachromosomal DNA were extracted from isolated hAFs for analysis with qRT‐PCR. To compare the PGE2 production in hAFs between TL and TNL groups, the conditional culture medium of hAFs was collected for analysis with ELISA.

### Reagents and Plasmids

Puromycin (#S9361), RU521 (#S6841), H‐151 (#S6652), Lamivudine/3TC (#S1706), Tenofovir (#S1401), and Etravirine (#S3080) were purchased from Selleckchem. rhS100A9 was purchased from R&D systems (#9254‐S9‐050). The adenovirus vectors carrying human genes including full‐length S100A9 in the nucleus (pDC315‐3NLS‐S100A9‐3NLS‐3Flag), full‐length CBX5 (pDC315‐CBX5‐SV40), wide type full‐length S100A9 (pDC315‐S100A9‐3Flag‐SV40‐EGFP), site‐directed mutagenesis (T113A) of full‐length S100A9 (pDC315‐S100A9T113A‐3Flag‐SV40‐EGFP), site‐directed mutagenesis (T113D) of full‐length S100A9 (pDC315‐S100A9T113D‐3Flag‐SV40‐EGFP) were prepared by Shanghai GeneChem, Co. Ltd. The “empty” vectors (pDC315‐3NLS‐3Flag, or pDC315, or pDC315‐3Flag‐SV40‐EGFP) were used for negative control where applicable. The scAAV vector expressing mouse full‐length S100A9 (posCAAV‐CMV‐S100A9‐3NLS‐3Flag‐tWPA) in the nucleus was constructed by Shanghai OBiO Technology. A corresponding “empty” vector (pCsCAAV‐CMV‐3NLS‐3Flag‐tWPA) was used as a negative control. NLS sequences (DPKKKRKV) were copied from Invitrogen's pCMV/myc/nuc. The efficiency of overexpression and subcellular localization was examined with western blotting or immunofluorescence staining in the corresponding experiment. The information on antibodies used in this study is given in Table S5, (Supporting Information).

### Adenovirus Infection and siRNA Transfection in hAFs

The hAFs isolated from the TL amnion were used for the S100A9 knockdown experiment, whereas the hAFs isolated from the TNL amnion were used for all other mechanistic studies to avoid the confounding effects of labor. For adenovirus infection, isolated hAFs were cultured for 24 h in Dulbecco's Modified Eagle Medium (DMEM; Life Technologies Inc.; #21063029) containing 10% fetal bovine serum (FBS; Biological Industries; #04‐121‐1A) and antibiotics at 37 °C in 5% CO_2_/95% air. Then, the cells were inoculated with adenovirus (1 × 10^9^ TU/mL DMEM) with packaging plasmids carrying NLS‐S100A9, CBX5, wild‐type S100A9, or T113A mutant S100A9, T113D mutant S100A9 for 24 h. Afterward, the solution was removed, and the cells were washed with PBS and incubated with complete DMEM for 48 h in the presence or absence of RU521 (1 µM), H151 (1 µM), 3TC (10 µM), Tenofovir (5 µM), or Etravirine (5 µM) before harvesting. For siRNA transfection, isolated hAFs were immediately incubated with 50 nM of two sets of siRNA against *CBX5* (1#: 5′‐GCCGAUGACAUCAAAUCUATT‐3′; 2#: 5′‐GGUGAUUUAAUGUUCCUAATT‐3′) or two sets of siRNA against *S100A9* (1#: 5′‐GCUUCGAGGAGUUCAUCAUTT‐3′; 2#: 5′‐GCAACAUAGAGACCAUCAUTT‐3′) or randomly scrambled siRNA (5′‐UUCUCCGAACGUGUCACGUTT‐3′; GenePharma) in Opti‐MEM (Life Technologies Inc.; #31985062) using an electroporator (Nepa Gene) at 165 V for 5ms. The cells were then incubated in complete DMEM for 72 h before harvesting for further processing and analysis. To investigate the effect of extracellular S100A9 on cellular senescence in hAFs, the cells were treated with rhS100A9 (0.05, 0.5, and 5 µg mL^−1^) for 24, 48, and 72 h 3 days after plating.

### RNA Extraction and qRT‐PCR

Total RNA was extracted from the snap‐frozen amnion tissue or hAFs with a total RNA isolation Kit (Foregene; #RE‐03113) according to the protocol provided by the manufacturer. After examination of RNA quality and concentration, reverse transcription was carried out using a Prime‐Script RT Master Mix Kit (TaKaRa; RR036B). The mRNA abundance of *S100A9, IFNA, IFNB, CDK1, E2F1, CCNE2, CDKN1A, PTGS1, PTGS2, OAS1, IFIT1, ISG15, CCL4, CXCL10*, *CBX5*, HERV‐K, and LINE1 was determined with qRT‐PCR using the above reverse‐transcribed cDNA and MagicSYBR Mixture (CoWin Biosciences; #CW3008). The housekeeping gene *GAPDH* (Glyceraldehyde 3‐phosphate dehydrogenase) was amplified in parallel as an internal control. The relative mRNA abundance was quantitated using the 2‐^△△Ct^ method, which was normalized to *GAPDH*. The primer sequences used for qRT‐PCR are illustrated in Table S6, (Supporting Information).

### Total, Nuclear and Cytoplasmic Protein Extraction

Total cellular protein was extracted from the snap‐frozen amnion tissue or hAFs with an ice‐cold radioimmunoprecipitation assay lysis buffer (WeiaoBio; #WB0101) containing protease inhibitor (Roche; #04693124001) and phosphatase inhibitor (Roche; #04906837001). Nuclear and cytoplasmic protein was extracted from the snap‐frozen amnion tissue, or hAFs using a MINUTE Cytoplasmic & Nuclear Extraction Kit (Invent Biotechnologies; #NT‐032) or a Nuclear Extract Kit (Active Motif; #40010) according to the respective protocol provided by the manufacturer.

### Western Blotting

After determination of protein concentration using the Bradford method, the abundance of S100A9, p‐S00A9 (Thr 113), p53, p21, COX‐2, H3K9me3, Flag, CBX5, ORF1p, ORF2p, IRF3, p‐IRF3 (Ser 396), TBK1, p‐TBK1 (Ser 172), STING, p‐STING (Ser 366) protein in each sample was determined with Western blotting. Briefly, 30 µg of protein from each sample was electrophoresed in a sodium dodecyl sulfate (SDS)‐polyacrylamide gel. After transferring to a nitrocellulose membrane blot (Millipore; #HATF00010), the blot was blocked with 5% non‐fat milk and incubated with primary antibodies overnight at 4 °C, followed by incubation with corresponding secondary antibodies conjugated with horseradish peroxidase (Proteintech). The peroxidase activity was developed with a chemiluminescence detection system (Millipore; WBKLS0500), which was visualized with the digital imaging equipment eBlot Touch Imager (eBlot Tec.). GAPDH, α‐tubulin, and Lamin A/C were used as internal control for total protein, cytoplasmic, and nuclear protein, respectively. Total H3 was used as an internal control for H3K9me3. The ratio of the phosphorylated protein to the corresponding total protein was used to indicate the relative abundance of phospho‐proteins. Information on antibodies used is given in Table S5 (Supporting Information).

### Immunohistochemical Staining

After deparaffinization of the paraffin‐embedded sections, antigen retrieval, and quenching of the endogenous peroxidase activity with 0.3% H_2_O_2_, the sections were blocked with non‐immune serum, followed by incubation with the corresponding primary antibody overnight at 4 °C. Pre‐immune IgG served as negative control. The antibody information is given in Table S5, Supporting Information. After washing, a biotinylated secondary antibody (Vector Laboratories; #BA‐1300) and the avidin‐biotin complex conjugated with horseradish peroxidase (Vector Laboratories; #PK‐7100) were applied to the sections for further incubation. Afterward, the peroxidase activity was developed as a red color using the substrate 3‐amino‐9‐ethyl carbazole (Vector Laboratories; #SK‐4200). The slide was counterstained with hematoxylin (blue color) and examined under bright field with a microscope (Zeiss; Axio Vert.A1). The percentage of p21‐positive cells was counted in five randomly selected visual fields with each field containing > 25 cells for each sample with ImageJ software (NIH).

### Immunofluorescence Staining

After deparaffinization of the paraffin‐embedded sections, antigen retrieval was performed. The sections were then permeabilized with 0.4% Triton X‐100. After blocking with normal goat serum, the corresponding primary antibodies were applied at 4 °C for incubation overnight, followed by incubation with Alexa Fluor 488‐, Alexa Fluor 594‐, or Alexa Fluor 647‐labeled secondary antibodies (Proteintech). Nuclei were counterstained with DAPI (blue color). For immunofluorescence staining in hAFs, the cells were fixed in 10% paraformaldehyde, and then primary antibodies against Vimentin, Cytokertin‐7, γ‐H2AX, Flag, p21, H3K9me3, S100A9 and p‐S100A9 (Thr 113) where applicable were applied. Nuclei and cytoskeleton were counterstained with DAPI (blue color) and phalloidin (red color) respectively. A confocal microscope system (TCS SP8; Leica) or a fluorescence microscope (Zeiss; Axio Vert.A1) was used to observe immunofluorescence signals. Quantification and statistical analysis of fluorescence signals were based on five random visual fields with each field containing > 25 cells for each sample with ImageJ software (NIH). The 3D reconstruction of H3K9me3 staining was performed by serial z‐stack sectioning for up to 80 images at 100‐nm intervals, and further processed to perform 3D reconstruction by using a Leica confocal system (TCS SP8). Exposure settings were unchanged throughout the acquisition of corresponding photomicrographs. The information of antibodies used in immunofluorescence staining is listed in Table S5 (Supporting Information).

### ELISA

The supernatant collected from the homogenized amnion tissue or the conditioned culture medium of hAFs was used for measurement of S100A9 or PGE2 where applicable with a S100A9 ELISA kit (R&D systems; #DY5578) or a PGE2 ELISA kit (Cayman Chemical; #514010) following the protocol from the manufacturer. The level of 2′3′‐cGAMP in the cell lysate of hAFs was measured using a 2′3′‐cGAMP ELISA Kit (Cayman Chemical; #501700) according to the manufacturer's instruction.

### Transcriptomic Sequencing

Total RNA was extracted from hAFs with or without nuclear S100A9 overexpression (n = 3 each) using Trizol reagent (Thermo Fisher Scientific; #15596026). RNA purity and integrity were determined using a NanoDropND‐2000 (Thermo Fisher Scientific) and an Agilent 2200 TapeStation. Transcriptomic sequencing libraries were constructed using a TruSeq RNA sample preparation kit (Illumina) according to the manufacturer's protocol, followed by sequencing of the libraries on an Illumina NovaSeq 6000 system. A computational pipeline was used to process the transcriptomic sequencing data. After removing the adaptor and low‐quality reads, the clean reads were aligned to the human genome (NCBI; GRCh38) using Hisat v.2.1.0 with default options.^[^ ^38^ ^]^ The fragment per kilobase per million mapped reads (FPKM) was obtained to indicate the abundance of transcripts. Differential expression was analyzed using the DESeq2 algorithm.^[^ ^39^ ^]^ *P* value < 0.05 and fold Change ≥1.5 or ≤0.67 were set as the threshold for significantly differential expression. GO and KEGG pathway enrichment analyses of differentially expressed genes were performed using R based on the hypergeometric distribution. GSEA (version 4.2.3) was used to statistically calculate the difference of a set of genes. Gene sets were obtained from the GSEA molecular signature database: https://www.gsea‐msigdb.org/gsea/msigdb.

### SA‐β‐Gal Staining Assay

The SA‐β‐gal staining was conducted using a commercial staining kit (Beyotime; #C0602). Briefly, after washing with PBS, the cryosections or cultured hAFs were fixed with 4% paraformaldehyde for 15 minutes at room temperature, and subsequently incubated with a working solution containing 0.05 mg mL^−1^ 5‐bromo‐4‐chloro‐3‐indolyl‐β‐d‐galactopyranoside at pH 6.0 overnight in darkness at 37 °C. For cryosections, nuclear fast red (Beyotime; #C0151) counterstaining was conducted for better visualization of cellular morphology. A phase contrast microscope system (Zeiss; Axio Observer 3) was used to capture the images. The percentage of SA‐β‐gal positive cells was counted in five random visual fields with each field containing > 25 cells in tissue staining and > 50 cells in cell staining for each sample with ImageJ software (NIH).

### LC‐MS/MS Analysis

Total protein was extracted from hAFs with or without nuclear S100A9 overexpression using IP lysis buffer (Thermo Fisher Scientific; #87787) containing protease and phosphatase inhibitors. After determining the protein concentration, 500 µg of protein was incubated with Flag antibody‐coupled beads (Selleckchem; #B26101) on a rotating platform overnight at 4 °C. The beads were then thoroughly washed, and the bound protein was eluted using glycine‐HCl (pH 2.5). Then the protein was reduced with 10 mM dithiothreitol and alkylated with 50 mM iodoacetamide, followed by acetone precipitation overnight. After centrifugation and washing with 90% acetone, the cleaned precipitates were dissolved with 50 mM ammonium bicarbonate and digested by trypsin at 37 °C overnight. The hydrolysates were desalted by C18 stage tips and dried using a vacuum drier. After re‐dissolving by 99.9% water with 0.1% formic acid, the hydrolysates were applied to a sample vial for LC‐MS analysis, which was performed on an EASY‐ nLC 1000 ultra‐high‐pressure system equipped with an Orbitrap Fusion Tribrid mass spectrometer (all from Thermo Fisher Scientific). After separation at 55 °C using a 25 cm × 75 µm C18 column (Aurora Ultimate; #AUR3‐25075C18‐CSI), the resulting peptides were subject to MS analysis in data‐independent acquisition (DIA) mode, which consisted of MS1 scan ranging from 390 to 980 m/z at 60000 resolution and the automatic gain control (AGC) target 1 × 10^6^ or a maximum injection time of 100 ms. Then, 27 DIA rolling segments with isolation windows of 17, 23, 29, and 35 m/z were acquired at the resolution of 30000 with an AGC target 5 × 10^5^ or a maximum injection time of 40 ms. The setting “injections for all available parallelizable time” was enabled, and higher‐energy collision dissociation fragmentation was performed with a normalized collision energy of 24%. The spectra were recorded in profile mode, with the default charge state for MS2 set to two. Then, the raw data were searched against an in silico predicted spectral library generated from UniProt human protein database with a default setting. Match‐between‐runs were employed using the spectral library created from DIA data, with precursor filtering at 0.01 of false discovery rate. Neural network classifier = double‐pass mode, Quantification strategy = robust high precision, Cross‐run normalization = RT‐dependent.

### Molecular Docking

HDOCK SERVER (http://hdock.phys.hust.edu.cn/) or ZDOCK SERVER (http://zdock.wenglab.org) was used for the molecular docking study between S100A9 and CBX5. S100A9 (Uniprot ID: P06702) was selected as the receptor protein and CBX5 (Uniprot ID: P45973) was selected as the ligand‐protein. Pre‐treatment of the protein (deletion of water molecules and excess ligands, addition of hydrogen atoms) was completed using PyMol 2.4. The model with the lowest binding energy was selected as the best docking model, and PyMOL was used to visualize protein‐protein interactions.

### Co‐IP Assay

Co‐IP assay was performed as previously described.^[^ ^40^ ^]^ Cellular protein was extracted from hAFs with or without nuclear S100A9 or T113D mutant S100A9 overexpression using IP lysis buffer (Thermo Fisher Scientific; #87787). After the determination of protein concentration, 500 µg of protein was incubated with Flag antibody‐coupled beads (Selleckchem; #B26101) on a rotating platform overnight at 4 °C. In parallel, another 500 µg of protein was incubated with antibodies against Flag (Cell Signaling Technology; #14793), S100A9 (Abcam; #ab63818), CBX5 (Cell Signaling Technology; #2616), or MBD3 (Proteintech; #14258‐1‐AP), or pre‐immune rabbit IgG as a negative control, and rotated at 4 °C overnight, followed by incubation with protein A agarose beads for 2 h at 4 °C with rotation to pull down the antibody/antigen complex. The precipitated beads were washed adequately and then denatured in Western blotting loading buffer at 100 °C for subsequent analysis with Western blotting.

### TEM Examination

TEM examination was performed to examine the heterochromatin disruption in hAFs with or without nuclear S100A9 overexpression. After rapid fixation with 2.5% glutaraldehyde at 4 °C for 2 h, and post‐fixation with 1% osmium tetroxide solution for 2 h at 4 °C, the cells were processed for dehydration, infiltration, and routine embedding in resin. After solidification, ultrathin sections (70 nm) were cut and stained with lead citrate, which was then observed for heterochromatin at the nuclear periphery with a transmission electron microscope (FEI Talos L 120C; Thermo Fisher Scientific).

### CUT&Tag

A Hyperactive Universal CUT&Tag Assay kit (Vazyme; #TD904) was used to construct the CUT&Tag libraries of hAFs with or without nuclear S100A9‐overexpression according to the manufacturer's protocol. In brief, 0.1 million cells for each sample were collected, washed, and resuspended in 100 µL wash buffer, and then 10 µL of activated concanavalin A‐coated magnetic beads were added to each sample and incubated at room temperature for 10 min. The cells were then incubated with 2 µg H3K9me3 antibody (Epigentek; #A‐4036‐025) on a rotating platform overnight at 4 °C, followed by incubation with an anti‐rabbit IgG antibody at room temperature for 1 h. After washing with Dig‐Wash buffer, the samples were incubated with pA/G‐Tnp, and resuspended in tagmentation buffer for fragmentation. Afterward, 2 µL 10% SDS and 1 pg Spike‐in DNA were added to each sample for normalization and incubated at 55 °C for 10 min. The fragmented DNA in the supernatant was then extracted with DNA Extract Beads Pro, and DNA libraries were constructed using TruePrep Index Kit V2 for Illumina (Vazyme; #TD202). All CUT&Tag libraries were sequenced using the NovaSeq X Plus platform with a PE150 sequencing strategy.

For the processing of H3K9me3 CUT&Tag data, raw reads were trimmed with Trim Galore software (version 0.4.5). Trimmed reads were mapped to the human genome (UCSC; hg38) using Bowtie2 (version 2.5.4).^[^ ^41^ ^]^ Duplicated reads were discarded by MarkDuplicates.jar program in Picard tools (version 1.119), and filtered quality reads were sorted with SAMtools (version 1.2.0).^[^ ^42^ ^]^ The sequencing reads were aligned to the spike‐in sequences using Bowtie2, and a normalization factor calculated as 10000 divided by the number of reads was applied to adjust all samples. MACS2 (version 1.1) was used to call peak with the parameters set to “‐f BAMPE –broad ‐q 0.05 –nolambda”. TE annotation file was downloaded from http://hgdownload.cse.ucsc.edu/goldenPath/hg38/database/rmsk.txt.gz. For the classification of H3K9me3‐enriched TEs regions, the H3K9me3 signals for each TE region were calculated. Then TEs regions were partitioned into “H3K9me3‐enriched TEs” and “other TEs” by robust k‐means analysis with two clusters using R package pheatmap (version 1.0.12). Analysis of differential H3K9me3 signals enriched in TEs regions were performed by Manorm (version 1.3.0), and the Log2 ratios of H3K9me3 signals for each H3K9me3‐enriched TEs were visualized by R package RIdeogram (version 0.2.2).

### ChIP Assay

The enrichments of H3K9me3, CBX5, MBD3, and KAP1 at LINE1 regions were determined with ChIP assay as described previously.^[^ ^40^ ^]^ Briefly, after crosslinking with 1% formaldehyde, hAFs were lysed with SDS lysis buffer containing a protease inhibitor cocktail (Roche). The lysed cells were sonicated to shear the chromatin DNA to an optimal size of around 500 bp, which was then immunoprecipitated with antibodies against H3K9me3, CBX5, MBD3, or KAP1. An equal amount of pre‐immune IgG served as negative control. Sheared DNA without immunoprecipitation served as input control. The immunoprecipitate was pulled down with Magna ChIP Protein A+G agarose Magnetic Beads (Millipore; #16‐663) on a magnetic stand. After reverse cross‐linking with 5 M NaCl, RNA and protein were digested with RNase A and proteinase K, respectively. Afterward, the sheared DNA was extracted using a DNA purification kit (Cwbiotech; #CWY012) for subsequent qRT‐PCR. The antibody information and primer sequences for ChIP‐qRT‐PCR are illustrated in Tables S5 and S7 (Supporting Information), respectively. The data were analyzed and presented as follows: percent input = 100% × 2^[(Ct (input sample)‐Ct (IP sample)^.

### LINE1 Retrotransposition Assay

LINE1 retrotransposition assay in hAFs was conducted following a modified protocol published previously.^[^ ^17^ ^]^ In brief, hAFs with or without nuclear S100A9 overexpression were transfected with 2 µg of the pEGFP‐LRE3 vector (a gift from Dr. Zhiyong Mao) using Lipofectamine 3000 (Thermo Fisher Scientific; L3000015). An empty vector served as the negative control. After transfection for 48 h the cells were incubated in DMEM containing 2 µg mL^−1^ puromycin for another 72 h. Then the surviving cells were harvested for flow cytometry analysis (BD Biosciences). The percentage of GFP‐positive cells was determined with the FlowJo software (Ashland).

### Extrachromosomal DNA Extraction

Extrachromosomal DNA was extracted using the modified Hirt protocol.^[^ ^43^ ^]^ After digestion from the culture plate, hAFs were resuspended in Buffer 1 (50 mM Tris‐HCl, 10 mM EDTA, containing 100 mg/mL RNase A), and then mixed and lysed with Buffer 2 (1.2% SDS) by inverting at room temperature for 5 min to ensure complete lysis. Afterward, cellular debris and high‐molecular weight chromosomal DNA were precipitated with addition of Buffer 3 (3 M CsCl, 1 M potassium acetate, and 0.67 M acetic acid) on ice for 15 min. The extrachromosomal DNA‐containing supernatant was then collected by centrifugation, and extrachromosomal DNA was purified using a DNA purification kit (Cwbiotech; #CWY012) according to the protocol provided by the manufacturer. Total cellular DNA was extracted from hAFs directly using the same DNA purification kit. The amount of LINE1 DNA content was determined with qRT‐PCR using the above extracted extrachromosomal DNA or total DNA as a template. Mitochondrial gene *MT‐ND5* (NADH dehydrogenase 5) and 5s rDNA were amplified in parallel as internal control for extrachromosomal DNA and total DNA, respectively.

### Animal Study

The mouse study was carried out following ARRIVE guidelines and the relevant ethical regulations for animal use, which was approved by the Institutional Review Board of Ren Ji Hospital, Shanghai Jiao Tong University School of Medicine (approval code: RJ‐2024‐28A). C57BL/6 mice (Charles River) of 10 to 13 weeks of age were mated overnight. The presence of a vaginal plug the next morning was counted as 0.5 dpc.

To investigate gestational age‐dependent changes in nuclear S100A9 and p21 protein, transcripts of LINE1 and mRNA associated with the type I IFN response and SASP, mouse fetal membranes were collected at 17.5 and 19 dpc (n = 5–6 per group). The abundance and distribution of nuclear S100A9 and p21 in the fetal membranes were determined with Western blotting and immunofluorescence staining using antibodies against mouse S100A9 and p21. The mRNA abundance of LINE1 and genes associated with the type I IFN response and SASP were determined with qRT‐PCR. The primers and antibodies information is illustrated in Tables S6 and S5 (Supporting Information), respectively.

To examine whether nuclear S100A9 was able to activate LINE1 resulting in the activation of the type I IFN response and cellular senescence in the fetal membranes thereby leading to preterm birth, intra‐amniotic injection of scAAV vectors expressing nuclear S100A9 (1 × 10^^10^ TU/10 µl/per gestational sac) in the presence or absence of Tenofovir (2 nmol/10 µl/per gestational sac) was performed at 15.5 dpc under laparotomy after anesthesia with 65 mg kg^−1^ Zoletil 50 (Virbac, #83887905). The same amount of empty scAAV vector served as a negative control. Some of the mice (n = 11 per group) were allowed to deliver spontaneously for observation of delivery time, and some (n = 6 per group) were sacrificed at 18 dpc (60 h after surgery) for collection of fetal membranes to examine the abundance of nuclear S100A9 and senescence phenotype. The abundance and distribution of nuclear S100A9 in the fetal membranes were determined with Western blotting and immunofluorescence staining respectively. The abundance of p21 in the fetal membranes was examined with Western blotting and immunohistochemical staining, and SA‐β‐gal staining was performed to detect the senescent cells. The transcripts of LINE1, mRNA associated with the type I IFN response, and SASP in the fetal membranes were measured with qRT‐PCR. The primers and antibodies information are illustrated in Tables S6 and S5 (Supporting Information), respectively. The abundance of PGE2 in the fetal membranes was measured with a PGE2 ELISA kit (Cayman; #514010) after extraction with ethyl acetate.

### Statistical Analysis

Data are presented as mean ± SEM. All the experiments were performed at least three times independently. The n number in each experiment provided in the figure legends represents independent experiments using samples from individual pregnant women or mice. The normality of the data was examined by Shapiro‐Wilk test. Based on the normality test, a two‐tailed paired or unpaired Student's t‐test or Mann‐Whitney U‐test was used to compare two groups where appropriate. One‐way ANOVA followed by Tukey's post hoc tests was performed when assessing the normally distributed data among three or more groups. Spearman correlation analysis was performed to test the correlation between groups. Methods of statistical analysis employed to determine the significance of difference in each experiment are also presented in related figure legends. Statistical significance was defined as *P* < 0.05.

### Ethics Approval Statement

The human study was conducted under a protocol approved by the Ethics Committee of Ren Ji Hospital, Shanghai Jiao Tong University School of Medicine with written informed consent from the participating patients (approval code: KY2023‐007‐B). All ethical regulations relevant to participants of human research were followed. The mouse study was carried out following ARRIVE guidelines and the relevant ethical regulations for animal use, which was approved by the Institutional Review Board of Ren Ji Hospital, Shanghai Jiao Tong University School of Medicine (approval code: RJ‐2024‐28A).

## Conflict of Interest

The authors declare no conflict of interest.

## Author Contributions

W.S.W, F.Z., and K.S. conceived the project and designed experiments. W.S.W and K.S. supervised the study. F.Z., M.D.L, F.P., and W.J.L. performed experiments. Y.X. contributed to LC‐MS/MS data collection and analysis. F.Z. and W.S.W. contributed to data analysis and interpretation. W.J.L. and L.J.L. collected samples from patients and analyzed clinical information. W.S.W, F.Z., L.M., and K.S. wrote the manuscript.

## Supporting information



Supporting Information

Supplemental Table 1

## Data Availability

Transcriptomic sequencing data from hAFs with or without nuclear S100A9 overexpression are available from the NCBI GEO accession GSE273966. CUT&Tag sequencing data from hAFs with or without nuclear S100A9 overexpression are available from the NCBI GEO accession GSE287835. LC‐MS/MS data from hAFs with or without nuclear S100A9 overexpression are available from the ProteomeXchange Consortium with the dataset identifier PXD054794. The data supporting the conclusions in this manuscript can be found in the main text or the Supplementary materials and are available from the corresponding author upon request.
