# Supplementary material for: Nuclear Translocation of S100A9 Triggers Senescence of Human Amnion Fibroblasts by De‐Repressing LINE1 Via Heterochromatin Erosion at Parturition
Source: Adv Sci (Weinh). 2025 Apr 2;12(21):2414682. doi: 10.1002/advs.202414682 (PMC12140318; doi:10.1002/advs.202414682)
Supplement: Supplementary file 1 — Supporting Information [file ADVS-12-2414682-s002.docx]

**Nuclear translocation of S100A9 triggers senescence of human amnion fibroblasts by de-repressing LINE1 via heterochromatin erosion at parturition**

Fan Zhang^1,2^, Meng-Die Li^1,2^, Fan Pan^1,2^, Wen-Jia Lei^3^, Yang Xi^4^, Li-Jun Ling^3^, Leslie Myatt^5^, Kang Sun^1,2^*, Wang-Sheng Wang^1,2^*

*Corresponding author. Email: wangsheng_wang@sjtu.edu.cn, or sungangrenji@sjtu.edu.cn

**This file includes:**

Figures S1 to S9

**Other Supplementary Materials for this manuscript include the following:**

Supplementary file 1: Tables S1 to S7

**
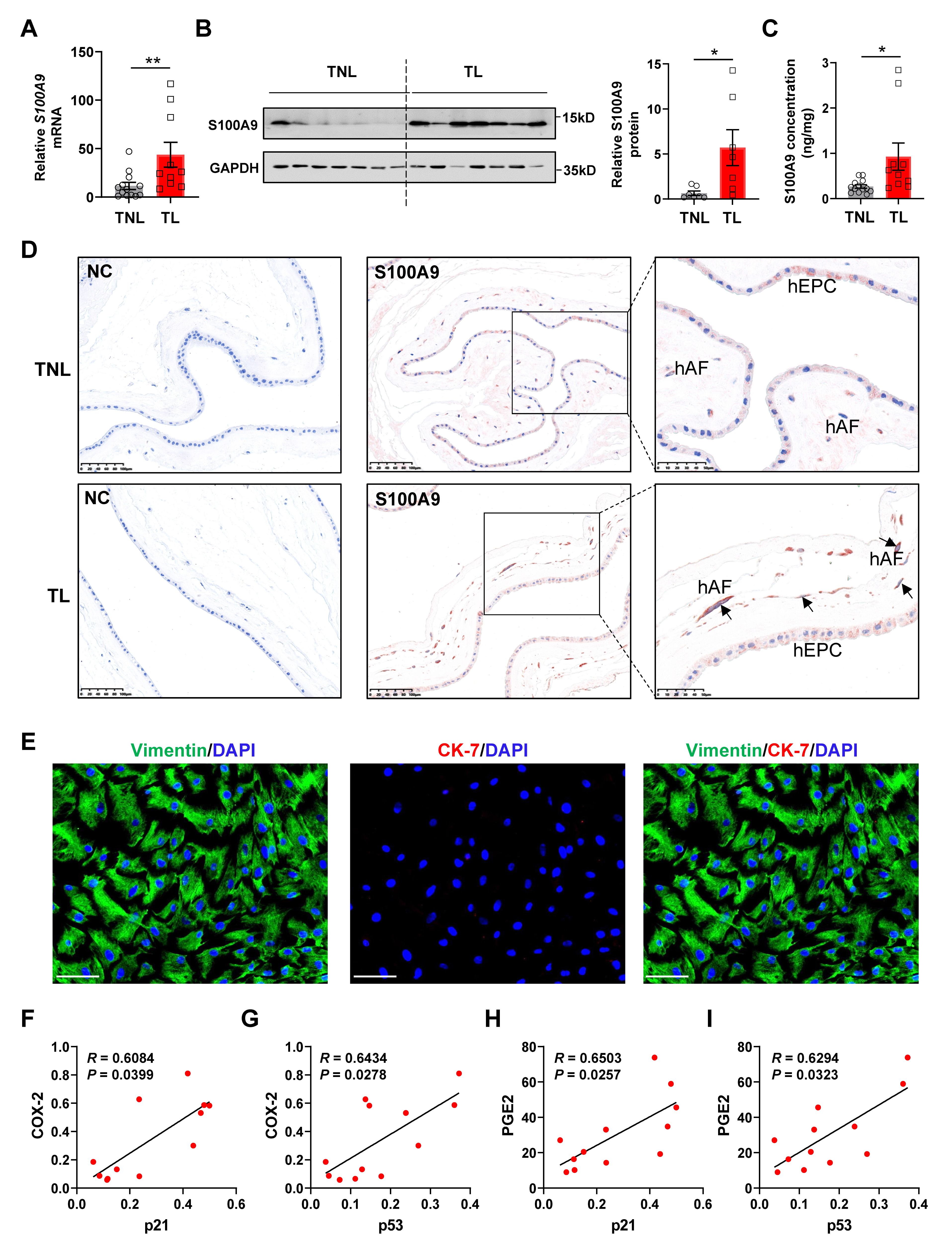
**

**Figure S1 Increased S100A9 protein abundance in human amnion at parturition. A-C**) qRT-PCR analysis (A), Western blotting (B) and ELISA (C) showing the difference of *S100A9* mRNA and S100A9 protein abundance in the human amnion between TL (n = 10 for A and C, n = 7 for B) and TNL (n = 13 for A and C, n = 7 for B) groups. Left panel of (B) is the Western blot and right panel of (B) is the average data. TL, term labor; TNL, term no labor. **D)** Representative immunohistochemical images showing intensive S100A9 staining (red color) in hAFs of the TL amnion. The tissue section was counterstained with hematoxylin (blue color). Black arrows indicate hAFs with S100A9 nuclear translocation. n = 3. Scale bars, 50 or 100 μm. NC, negative control. **E)** Immunofluorescent staining of cultured hAFs with antibodies against Vimentin (green color) and cytokeratin-7 (CK-7; red color). The nuclei were stained with DAPI (blue color). Vimentin and CK-7 are the representative markers for mesenchymal and epithelial cells, respectively. Scale bars, 100 μm. **F and G)** Spearman analysis showing positive correlation between COX-2 and p21 (F) or p53 (G) in isolated hAFs. n = 12. **H and I)** Spearman analysis showing positive correlation between PGE2 and p21 (H) or p53 (I) in isolated hAFs. n = 12. Data are mean ± SEM. Mann–Whitney U test (A-C). **p* < 0.05, ***p* < 0.01.


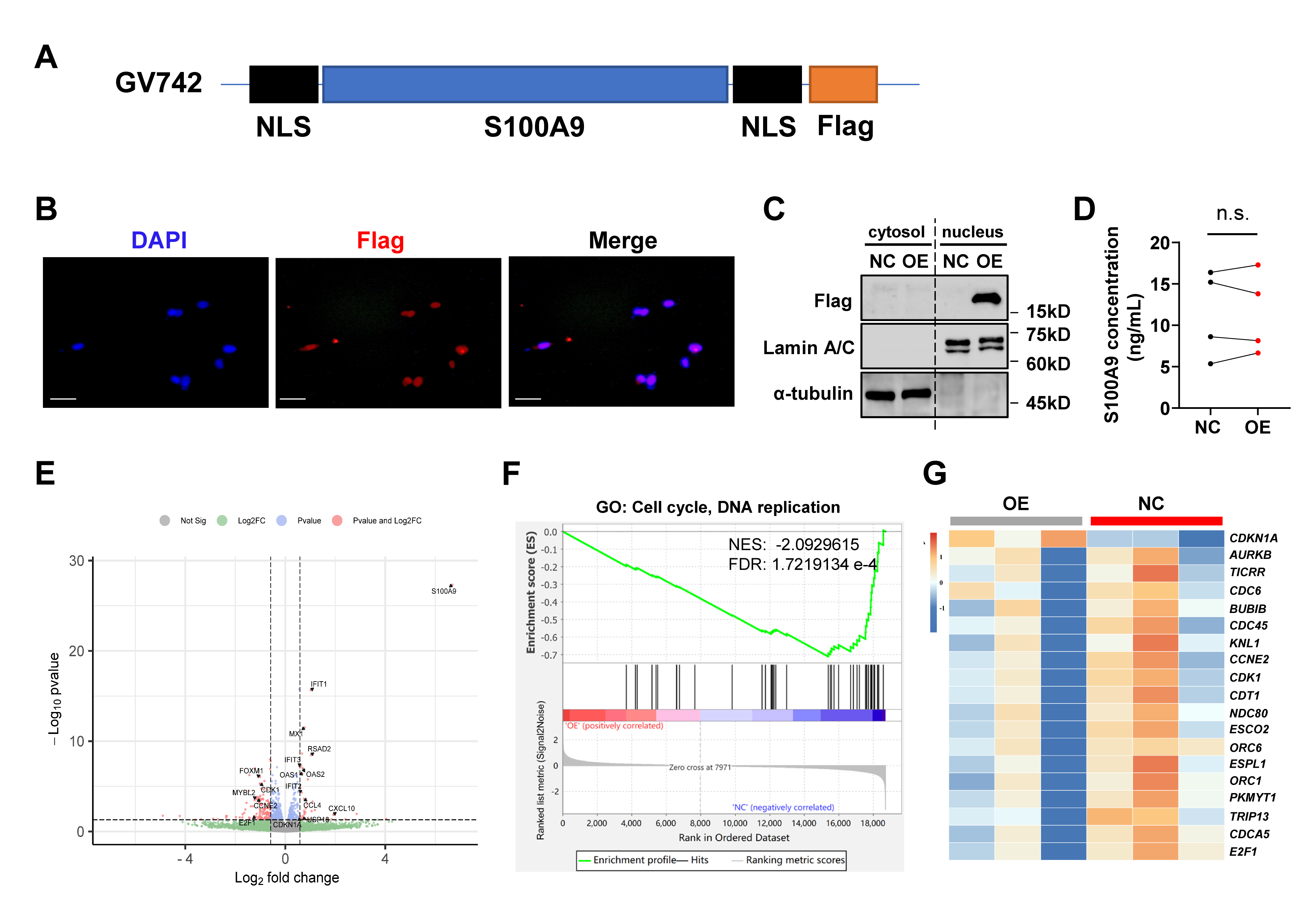


**Figure S2 Analysis of transcriptomic sequencing data obtained from hAFs with or without nuclear overexpression of S100A9. A)** The structure of adenovirus vector expressing the full-length of human S100A9 in the nucleus. **B and C)** Immunofluorescence staining of Flag (green color) (B) and Western blotting (C) showing the nuclear localization of S100A9-Flag. Nuclei were counterstained with DAPI (blue color). Scale bars, 50 μm. NC, control with empty vector transfection; OE, nuclear S100A9 overexpression. **D)** ELISA showing the abundance of S100A9 in the conditioned culture medium of hAFs with or without nuclear S100A9 overexpression. n.s., no significance. n = 4. **E)** Volcano plot of transcriptomic sequencing data (n = 3 per group) displaying the differentially expressed genes in hAFs with or without nuclear S100A9 overexpression. **F)** GSEA in GO enrichment showing that cell cycle, DNA replication pathways were downregulated in hAFs with nuclear S100A9 overexpression. NES, normalized enrichment scores; FDR, false discovery rate. **G)** Heatmap of transcriptomic sequencing data displaying the changed transcripts of gene enriched in KEGG pathways of cell cycle and cellular senescence. Blue to red represents expression levels from low to high. Data are mean ± SEM. Two-tailed paired Student’s t-test (D).


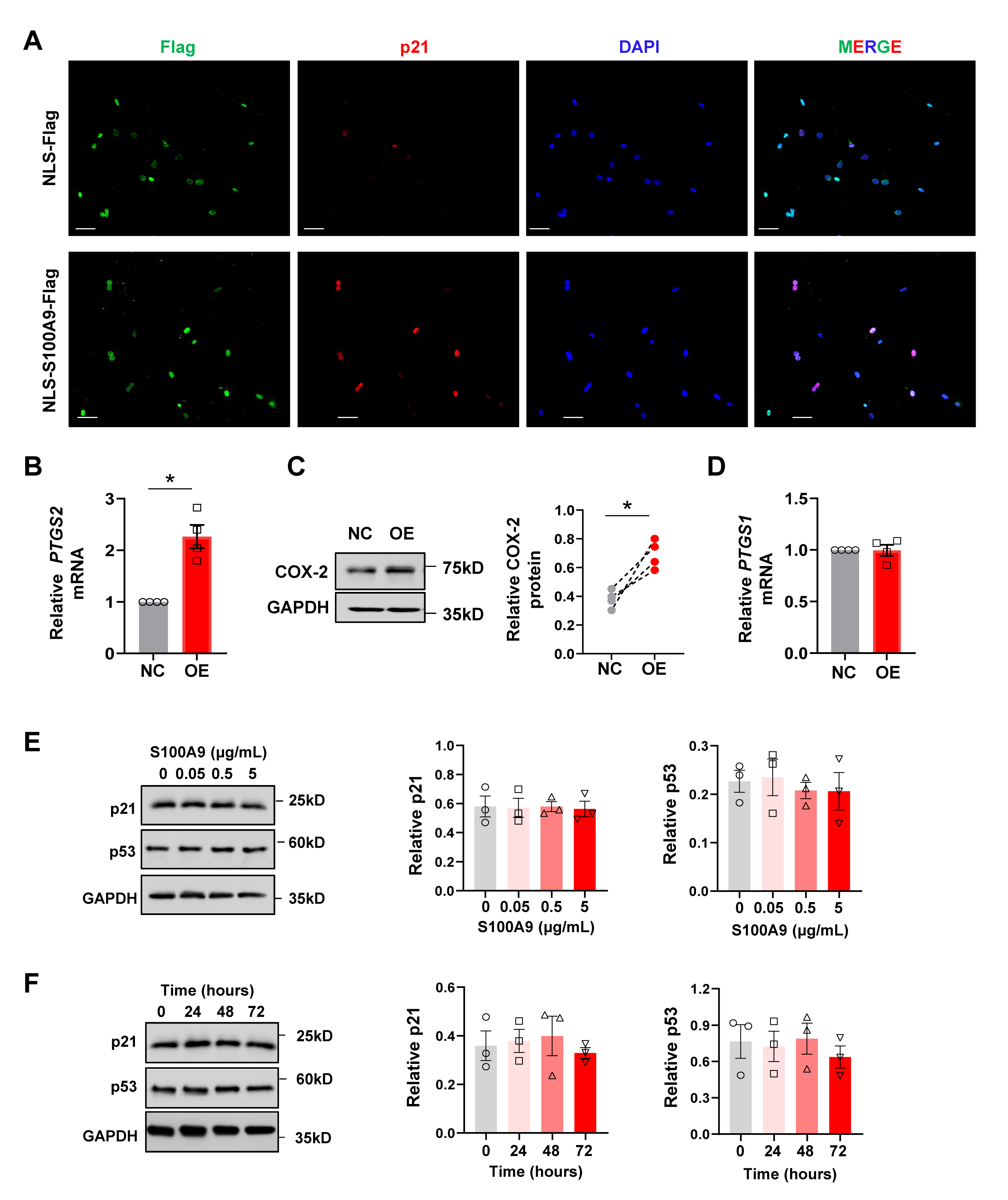


**Figure S3 Effect of nuclear and extracellular S100A9 on cellular senescence in hAFs. A)** Dual immunofluorescent staining of p21 (red color) and Flag (green color) in hAFs with or without Flag-S100A9 overexpression. Nuclei were counterstained with DAPI (blue color). Scale bars, 50 μm. n = 3. **B and C)** Abundance of *PTGS2* mRNA and COX-2 protein in hAFs with or without nuclear S100A9 overexpression. Left panel of (C) is the representative blot, and right panel of (C) is the average data. n = 4. **D)** Abundacne of *PTGS1* mRNA in hAFs with or without nuclear S100A9 overexpression. n = 4. **E)** Concentration-dependent effect of rhS100A9 (0.05, 0.5 and 5 μg/mL; 72 hours) on the protein abundance of p53 and p21 in hAFs. Left panel is the representative blot, and right panel is the average data. n = 3. **F)** Time-dependent effect of recombinant human S100A9 (rhS100A9; 0.5 μg/mL; 24, 48 and 72 hours) on the protein abundance of p53 and p21 in hAFs. Left panel is the representative blot, and right panel is the average data. n = 3. Data are mean ± SEM. Two-tailed paired Student’s t-test (B-D), or One-way ANOVA followed by Tukey's post hoc tests (E and F). **p* < 0.05.


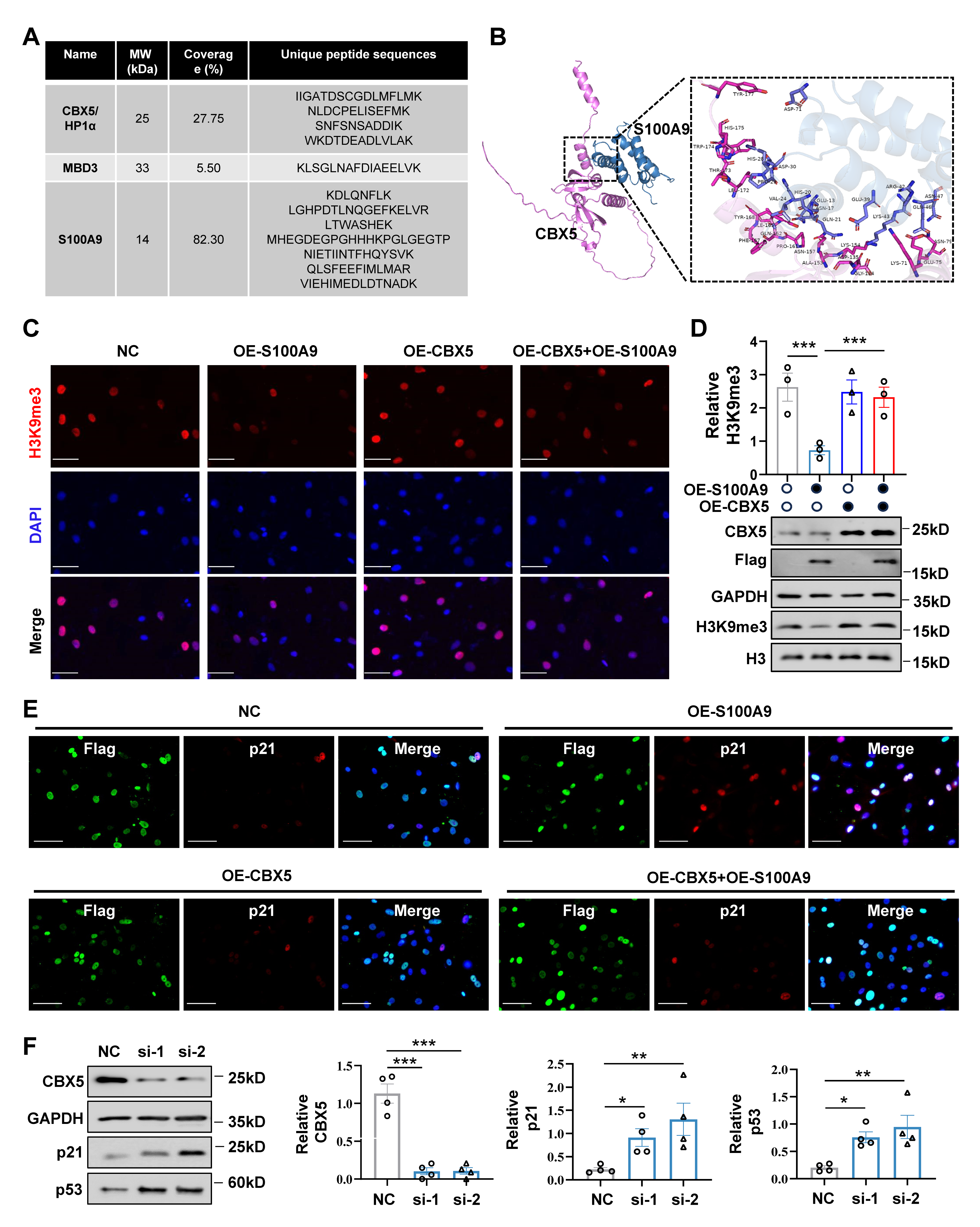


**Figure S4 Role of CBX5 in heterochromatin maintenance and cellular senescence in hAFs.** **A)** Detailed data illustrating the identification of CBX5 and MBD3 as S100A9 interacting candidates with mass spectrometry. **B)** Protein-protein docking prediction by H-DOCK SERVER showing overall co-complex structure of S100A9 (blue color) with CBX5 (purple color). Panel in the large box is the enlarged views of the binding site. **C and D)** Immunofluorescence staining (C, n = 4) and Western blotting analysis (D, n = 3) of H3K9me3 in hAFs with or without nuclear S100A9 overexpression in the presence or absence of CBX5 overexpression. Nuclei were counterstained with DAPI (blue color). Upper panel of (D) is the average data and lower panel of (D) is the representative blot. Scale bars, 50 μm. OE, overexpression. **E)** Dual immunofluorescent staining of p21 (red color) and Flag (green color) in hAFs with or without nuclear S100A9 overexpression in the presence or absence of CBX5 overexpression. Nuclei were counterstained with DAPI (blue color). Scale bars, 50 μm. **F)** Western blotting analysis showing the abundance of p53 and p21 in hAFs with or without siRNA-mediated knockdown of *CBX5*. Left panel is the representative blot and right panel is the average data. Two independent sets of siRNA against *CBX5* (si-1 and si-2) were used. n = 4. Data are mean ± SEM. One-way ANOVA followed by Tukey's post hoc tests (D and F). **p* < 0.05, ***p* < 0.01, ****p* < 0.001.


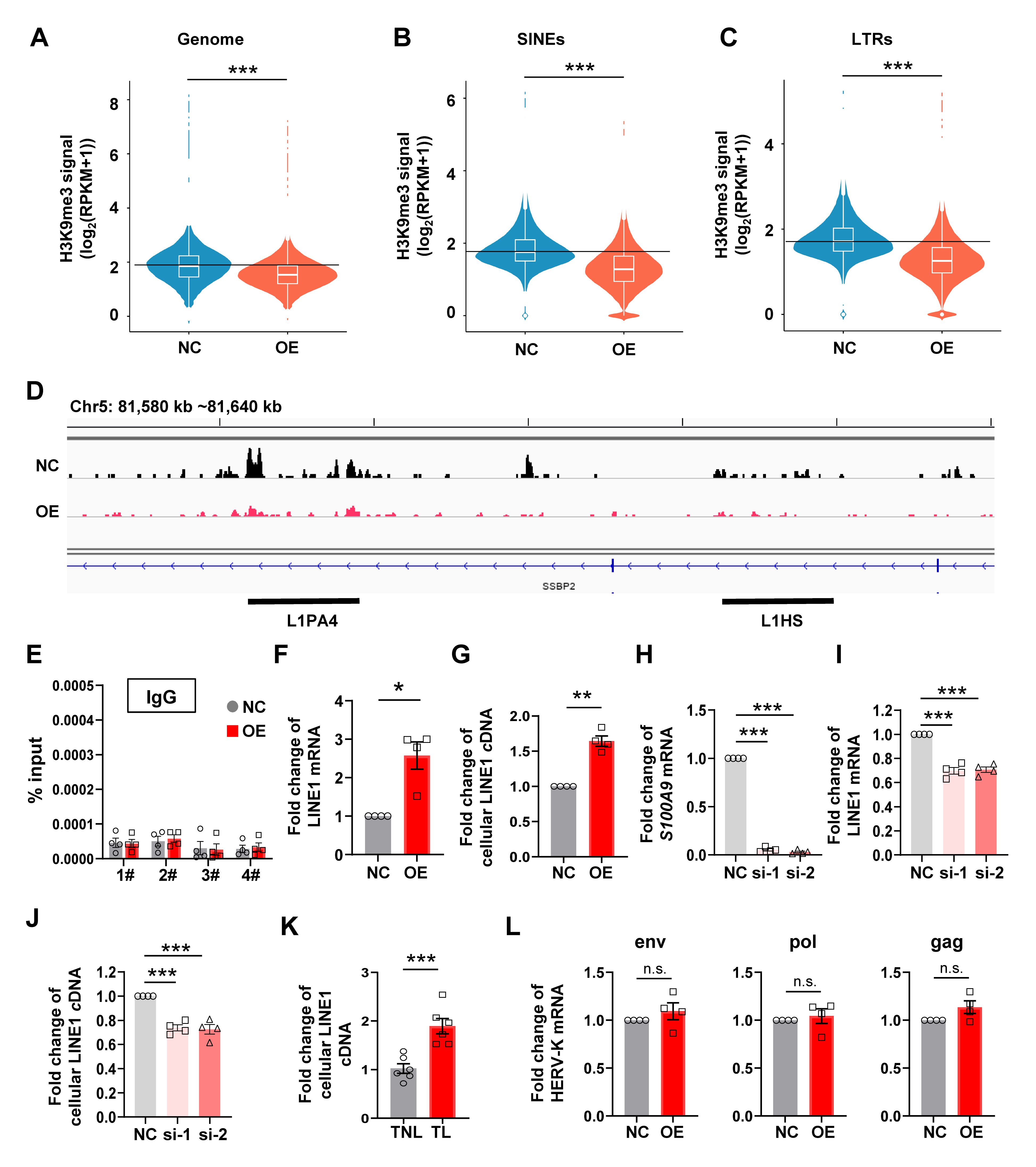


**Figure S5 LINE1 mRNA and cDNA contents in hAFs. A)** The violin plot showing H3K9me3 signals in overall genome region in hAFs with or without nuclear S100A9 overexpression. RPKM, Reads Per Kilobase per Million mapped reads. NC, control with empty vector transfection; OE, nuclear S100A9 overexpression. **B-C)** The violin plot showing H3K9me3 signals enriched in SINEs (B) and LTRs (C) in hAFs with or without nuclear S100A9 overexpression. **D)** A genome browser view for one example region with decreased H3K9me3 (red) over an evolutionarily young LINE-1s (L1HS and L1PA4) in hAFs with nuclear S100A9 expression. E**)** The negative control (pre-immune IgG) of Figure 4E. n = 4. **F)** qRT-qPCR with primers aligned to ORF2p showing LINE1 mRNA abundance in hAFs with or without nuclear S100A9 overexpressing. n = 4. Data with another primer set that aligned to ORF1p were shown in Figure 5F. **G)** Measurement with qRT-PCR showing total cellular LINE1 cDNA contents in hAFs with or without nuclear S100A9 overexpression. 5s rDNA was used as the reference gene. n = 4. **H and I)** Measurement with qRT-PCR showing *S100A9* (H) and LINE1 (I) mRNA abundance in hAFs with or without siRNA-mediated knockdown of *S100A9*. NC, negative control with scrambled siRNA; si-1 and si-2 are two separated siRNA against *S100A9*. Primers aligned to ORF1p (F). n = 4. **J)** Measurement with qRT-PCR showing total cellular LINE1 cDNA contents in hAFs with or without siRNA-mediated knockdown of *S100A9*. 5s rDNA was used as the reference gene. n = 4. **K)** Measurement with qRT-PCR showing total cellular LINE1 cDNA contents in hAFs isolated from TL (n = 6) and TNL (n = 6). 5s rDNA was used as the reference gene. **L)** The abundance of HERV-K mRNA in hAFs with or without nuclear S100A9 expression. Primers aligned to env, gas and pol respectively. n.s., no significance. n = 4. Data are mean ± SEM. Two-sided Wilcoxon signed-rank test (A-C), two-tailed paired (E, F, G, L) and unpaired Student’s t-test (K), One-way ANOVA followed by Tukey's post hoc tests (H-J). **p* < 0.05, ***p* < 0.01, ****p* < 0.001.


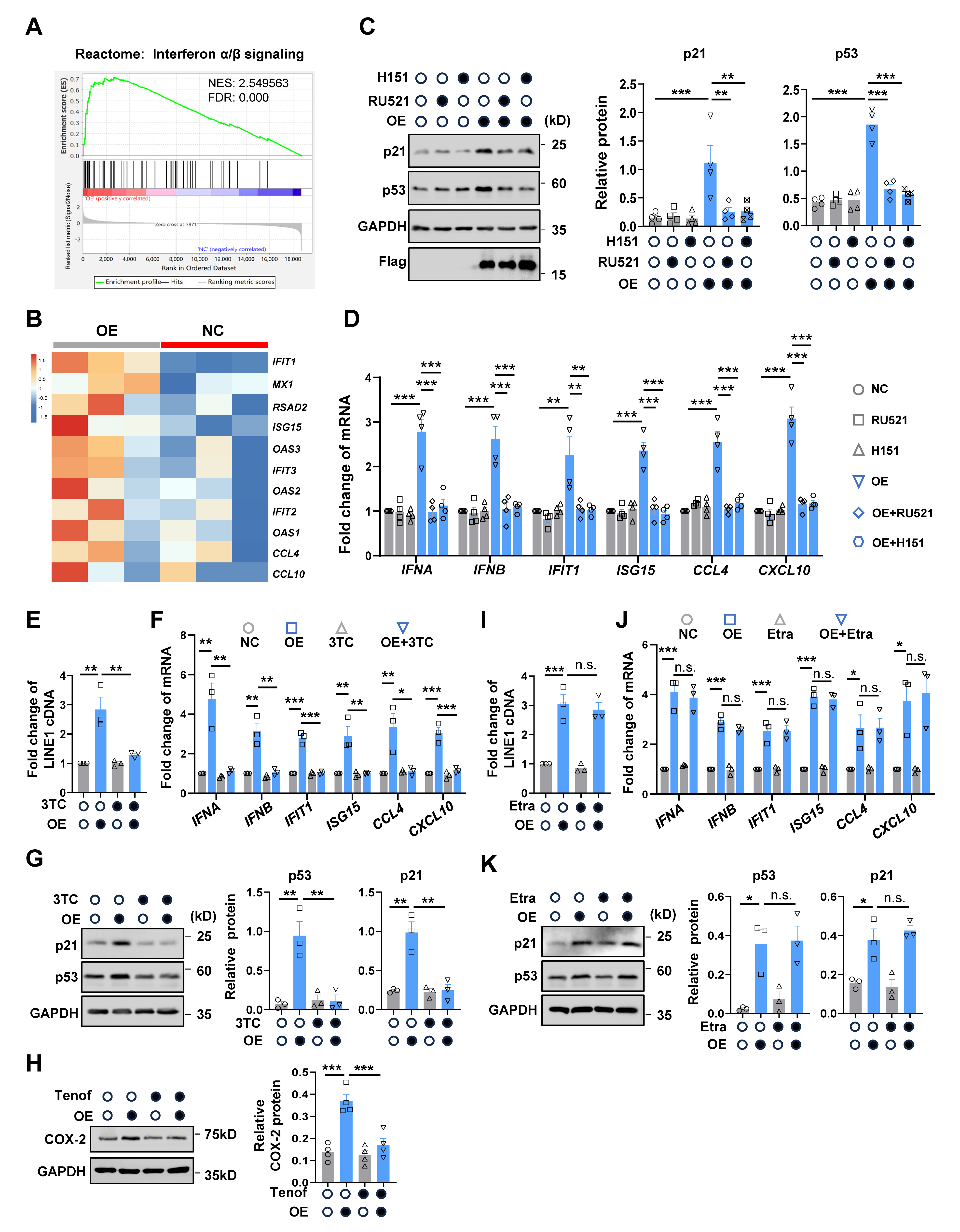


**Figure S6 Induction of the type I INF response and cellular senescence by nuclear S100A9 via activation of LINE1 in hAFs**. **A)** GSEA in Reactome pathway enrichment showing upregulation of the interferon α/β signaling in hAFs with nuclear S100A9 overexpression**.** NES, normalized enrichment scores; FDR, false discovery rate; NC, negative control with empty vector transfection; OE, nuclear S100A9 overexpression. **B)** Heatmap of transcriptomic sequencing data displaying the transcripts of gene enriched in Reactome pathway of interferon alpha/beta signaling. Blue to red represents expression from low to high. **C)** Western blotting analysis showing the protein abundance of p21 and p53 in hAFs with or without nuclear S100A9 overexpression in the presence or absence of RU521 and H151. Left panel is the representative blot, and right panel is the average data. n = 4. **D)** Measurement with qRT-PCR showing the abundance of mRNA associated with type I IFN responses and SASP in hAFs with or without nuclear S100A9 overexpression in the presence or absence of RU521 and H151. n = 4. **E and F)** Measurement with qRT-PCR showing the abundance of LINE1 cDNA contents (E) and mRNA associated with type I IFN response and SASP (F) in hAFs with or without nuclear S100A9 overexpression in the presence or absence of 3TC. n = 3. **G)** Western blotting analysis showing the protein abundance of p21 and p53 in hAFs with or without nuclear S100A9 overexpression in the presence or absence of 3TC. Left panel is the representative blot and right panel is the average data. n = 3. **H)** Western blotting analysis showing the abundance of COX-2 protein in hAFs with or without nuclear S100A9 overexpression in the presence or absence of Tenofovir (Tenof). Left panel is the representative blot, and right panel is the average data. n = 4. **I-K)** LINE1 cDNA contents (I), mRNA abundance associated with the type I IFN response and SASP (J), and p21 and p53 protein abundance (K) in hAFs with or without nuclear S100A9 overexpressing in the presence or absence of Etravirine (Etra). n = 3. Left panel of (K) is the representative blot and right panel of (K) is the average data. n.s. no significance. Data are mean ± SEM. One-way ANOVA followed by Tukey's post hoc tests (C-K). **p* < 0.05, ***p* < 0.01, ****p* < 0.001.


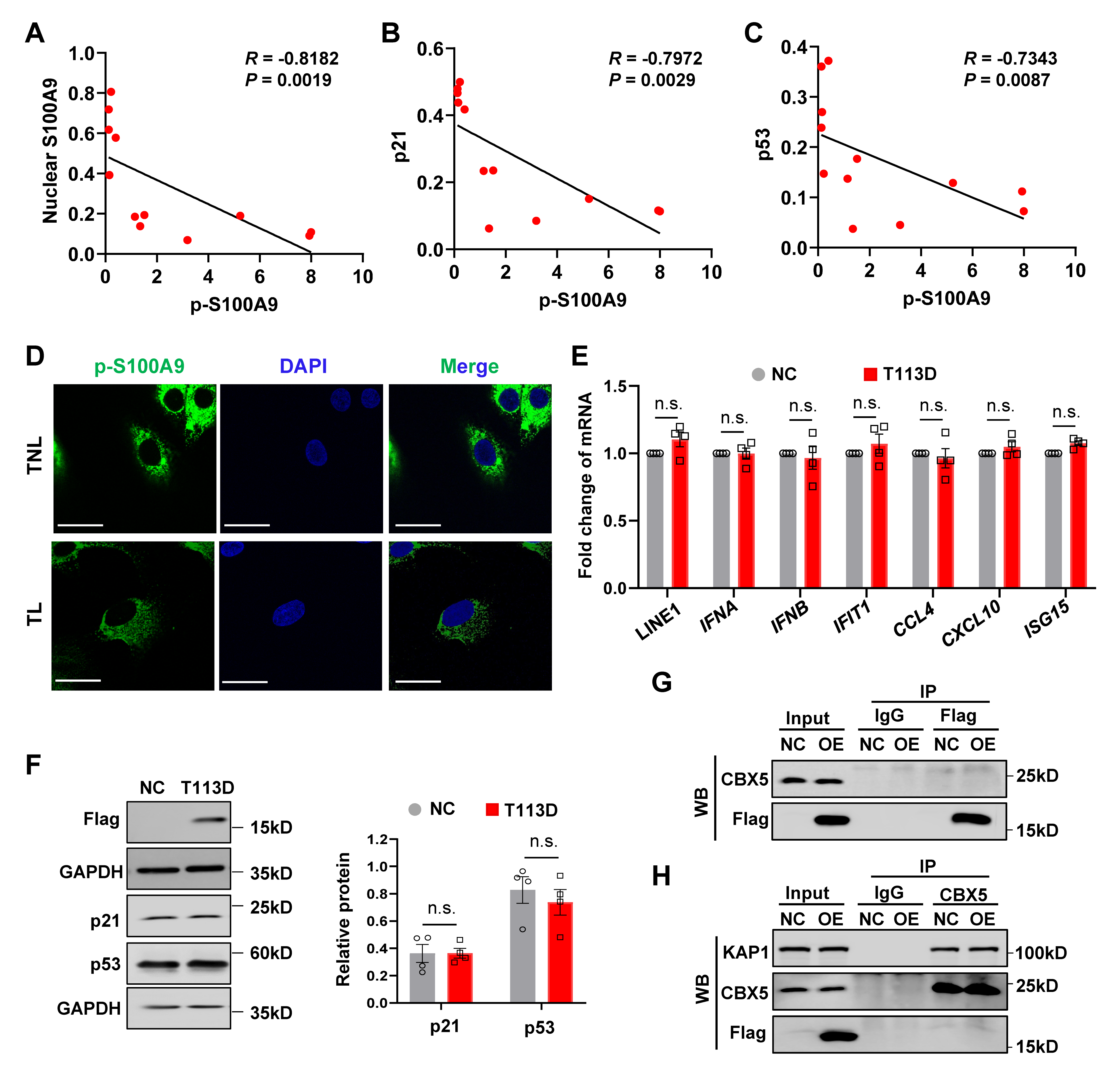


**Figure S7 Ineffectiveness of S100A9 with T113D (Thr→Asp) phosphorylation-mimic mutant on LINE1 activation, type I IFN response and cellular senescence in hAFs. A-C)** Spearman analysis showing negative correlation between p-S100A9 and nuclear S100A9 (A), p21 (B) or p53 (C) in isolated hAFs. n = 12. **D)** Representative image of immunofluorescent staining showing the intensified distribution of p-S100A9 (green color) in the cytoplasm of hAFs isolated from TL as compared to that from TNL pregnancies. Nuclei were counterstained with DAPI (blue color). n = 3. Scar bar, 25 μm. TL, term labor; TNL, term no labor. **E)** Measurement with qRT-qPCR showing the abundance of LINE1 and mRNA associated with the type I IFN response and SASP in hAFs with or without overexpression of S100A9 with T113D mutant. n.s. no significance. n = 4. **F)** Western blotting analysis showing the protein abundance of p53 and p21 in hAFs with or without overexpression of S100A9 with T113D mutant. n = 4. Left panel of (F) is the representative blots and right panel of (F) is the average data. n.s. no significance. **G and H)** Co-IP analysis showing the interaction between T113D mutant S100A9 and CBX5 (G), and the interaction between KAP1 and CBX5 (H) in hAFs with or without T113D mutant S100A9 overexpression. Input and pre-immune IgG served as positive and negative controls respectively. NC, negative control with empty vector; OE, T113D mutant S100A9 overexpression. n = 3. Data are mean ± SEM. Two-tailed paired Student’s t-test (E, F).


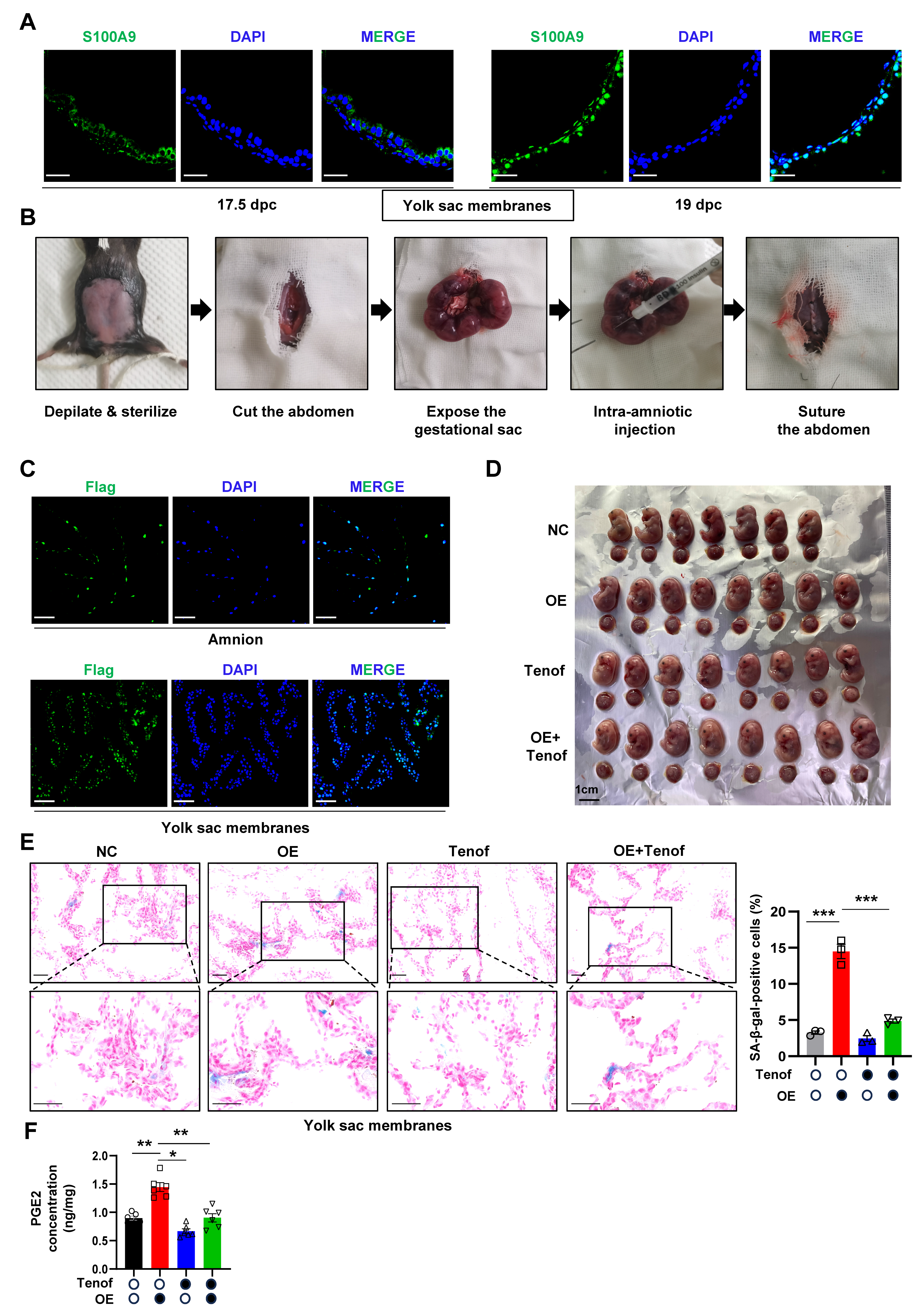


**Figure S8 Effects of intra-amniotic injection of scAAV vector expressing S100A9 in the nucleus in the mouse. A)** Representative images of immunofluorescence staining showing intensive S100A9 staining (green color) in cell nuclei of the yolk sac membranes at 19 dpc. Nuclei were counterstained with DAPI (blue color). Scale bars, 25 μm. n = 3 per group. **B)** The procedure of intra-amniotic injection in pregnant mice. **C)** Representative images of immunofluorescence staining of the Flag showing the nuclear localization of S100A9 (green color) upon intra-amniotic injection of scAAV vector expressing S100A9. Nuclei were counterstained with DAPI (blue color). Scale bars, 50 μm. **D)** Representative image showing the fetus and placenta of the same litter from the pregnant mice with intra-amniotic injection of scAAV vector expressing S100A9 in the nucleus in the presence or absence of Tenofovir (Tenof; 2 nmol/10 μl/per gestational sac). Scale bars, 1 cm. **E)** SA-β-gal staining of the yolk sac layer of the mouse fetal membranes with intra-amniotic injection of scAAV vector expressing S100A9 in the nucleus in the presence or absence of Tenofovir (Tenof; 2 nmol/10 μl/per gestational sac). Nuclei were counterstained with nuclear fast red (red color). n = 3 per group. Left panel is the representative image and right panel is the average data. Scale bars, 50 μm. NC, control empty vector. OE, scAAV vector expressing S100A9 in the nucleus. **F)** Measurement with ELISA showing PGE2 concentration in the mouse fetal membranes with intra-amniotic injection of scAAV vector expressing S100A9 in the nucleus in the presence or absence of Tenof (2 nmol/10 μl/per gestational sac). n = 6 per group. One-way ANOVA followed by Tukey's post hoc tests (E, F). **p* < 0.05, ***p* < 0.01, ****p* < 0.001.


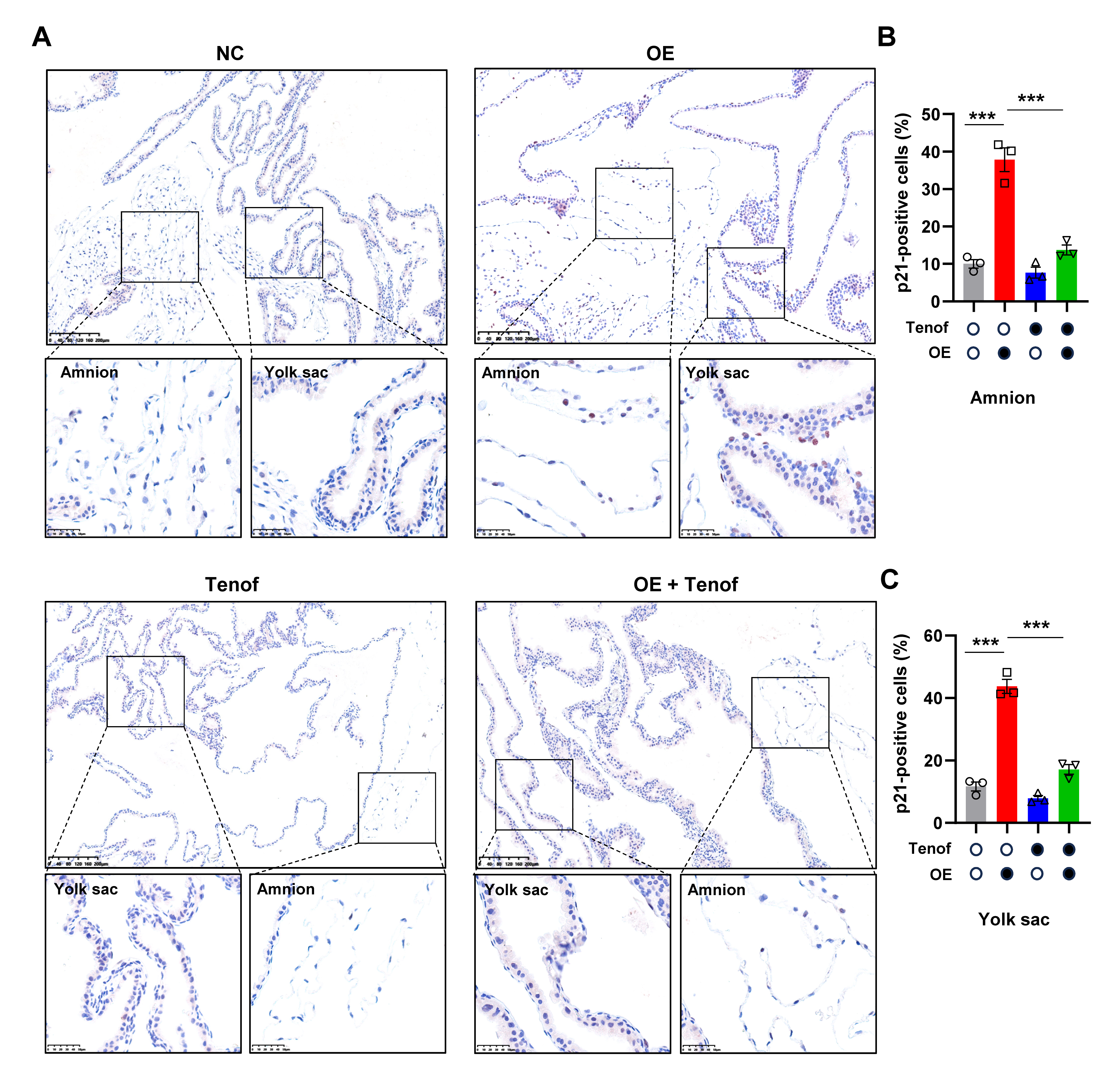


**Figure S9 Abundance of p21 in the mouse fetal membranes. A-C)** Immunohistochemical staining of p21 (red color) in the fetal membranes collected from mice with intra-amniotic injection of scAAV vector expressing nuclear S100A9 in the presence or absence of Tenofovir (Tenof; 2 nmol/10 μl/per gestational sac). The tissue section was counterstained with hematoxylin (blue color). (A) is the representative image, (B) and (C) are the average data. n = 3 per group. Scale bars, 50 μm or 200 μm. NC, control empty vector. OE, scAAV vector expressing S100A9 in the nucleus. One-way ANOVA followed by Tukey's post hoc tests (B, C). ****p* < 0.001.
